# Highly reactive 2-deoxy-2-iodo-d-*allo* and d-*gulo* pyranosyl sulfoxide donors ensure β-stereoselective glycosylations with steroidal aglycones†

**DOI:** 10.1039/c8ra06619a

**Published:** 2018-08-24

**Authors:** Jordi Mestre, David Collado, David Benito-Alifonso, Miguel A. Rodríguez, M. Isabel Matheu, Yolanda Díaz, Sergio Castillón, Omar Boutureira

**Affiliations:** Departament de Química Analítica i Química Orgànica, Universitat Rovira i Virgili C/Marcel·lí Domingo 1 43007 Tarragona Spain omar.boutureira@urv.cat

## Abstract

The preparation of well-defined d-*xylo* and d-*ribo* glycosides represents a synthetic challenge due to the limited configurational availability of starting materials and the laborious synthesis of homogeneous 2-deoxy-β-glycosidic linkages, in particular that of the sugar-steroid motif, which represents the “stereoselective determining step” of the overall synthesis. Herein we describe the use of 2-deoxy-2-iodo-glycopyranosyl sulfoxides accessible from widely available d-xylose and d-ribose monosaccharides as privileged glycosyl donors that permit activation at very low temperature. This ensures a precise kinetic control for a complete 1,2-*trans* stereoselective glycosylation of particularly challenging steroidal aglycones.

## Abstract 2

Highly stereoselective synthesis of challenging steroidal 2-deoxy-β-glycosides with d-*xylo* and d-*ribo* configurations enabled by low temperature activation of 2-deoxy-2-iodoglycopyranosyl sulfoxides.

## Graphical abstract

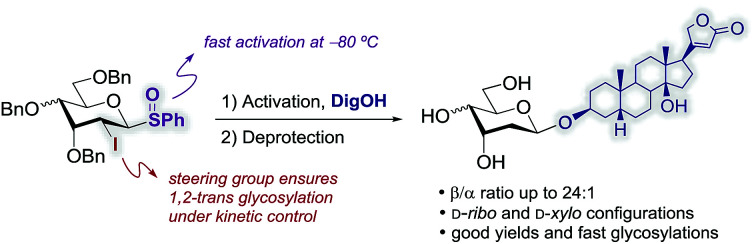

## Introduction

**Fig. 1:**
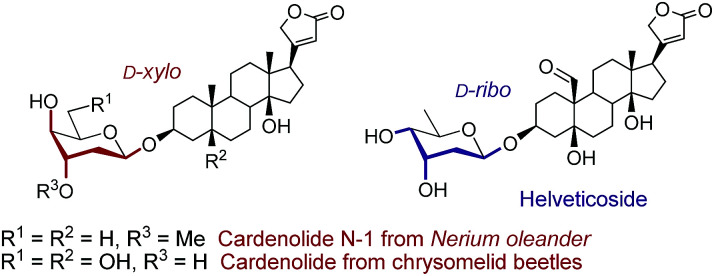
Naturally occurring 2-deoxy and 2,6-dideoxy-β-glycosides with “rare” d-*xylo* and d-*ribo* configurations.

**Scheme 1:**
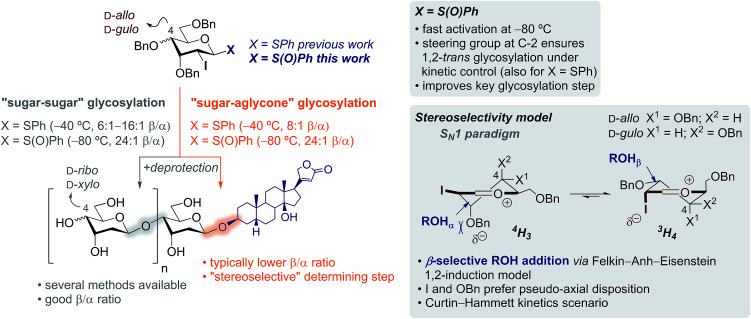
Scope and limitations of the stereoselective synthesis of β-steroidal glycosides of d-*ribo* and d-*xylo* configurations – using sulfoxides to improve key “sugar–aglycone” glycosylation step.

2-Deoxy- and 2,6-dideoxy-β-glycosides are common architectures present in many biologically active ingredients such as antibiotics, appetite suppressants, and nucleosides.^1^ These deoxyglycosides, and especially cardiac glycosides (*e.g.*, cardenolides N-1 from *Nerium oleander* or those from chrysomelid beetles,^3^ and helveticoside^4^), are usually composed of uncommon glycosyl moieties including d-*ribo* and d-*xylo*-configured pyranoses (Fig. 1). While these glycoconjugates, with the general structure [sugar]_*n*_–aglycone, are nicely produced in nature, most of the chemical glycosylation approaches^5^ for their preparation are mainly focused on the sugar–sugar motif but are still inefficient for the β-stereoselective synthesis of the sugar-steroid portion. In addition, the β-stereoselectivity is typically better for the construction of the sugar–sugar motif (up to only β) compared to that of the sugar-steroid fragment (up to 9 : 1 β/α) and thus, the latter glycosylation step represents the overall “stereoselective determining step” in 2-deoxy and 2,6-dideoxyglycoconjugates featuring such particular configurations.^5,6^ Our group has developed an indirect^7^ synthetic approach for the stereoselective synthesis of 2-deoxy- and 2,6-dideoxy-2-iodoglycosides that utilizes 2-deoxy-2-iodo-1-thioglycoside donors, being particularly effective for the production of β-d-*allo* and β-d-*gulo* pyranosides.^2,8–10^ The resulting configuration is predefined by the starting furanose and thus, d-*ribo* and d-*xylo* structures serve as configurational templates for d-*allo* and d-*gulo* pyranosides, respectively. Our findings determined the key β-selective glycosylation step is kinetically-controlled and the presence of iodine favours the stereoselective formation of a 1,2-*trans* glycoside *via* the least energetic transition state upon preferential nucleophilic attack to the oxonium intermediate ^3^H_4_. This is consistent with the Felkin–Anh–Eisenstein 1,2-induction model with stabilizing hyperconjugative interactions between 
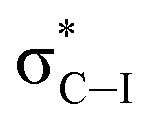
 and σ_C–OR_ (Scheme 1). According to most current models,^11^ the stereoselectivity is determined by the interplay between (a) the ground-state conformational preferences of oxocarbenium intermediates (^4^H_3_*vs.*^3^H_4_) in which electronegative substituents such as I and OBn prefer a pseudo-axial disposition due to stabilizing electrostatic and/or hyperconjugative interactions (*e.g.*, between σ_C–I_ and 
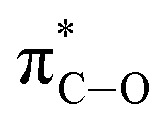
) and (b) the relative reactivity of each conformer under the S_N_1 paradigm, according to a Curtin–Hammett kinetics scenario. In this context, while glycosylations of 2-deoxy-2-iodo-1-thioglycosides with sugar acceptors proceed at *ca.* −40 °C and provided reasonably good selectivities (up to 16 : 1 β/α), we observed a reduction to 8 : 1 β/α with steroidal aglycones (Scheme 1).^8^ We reasoned that prior oxidation of the 1-thiophenyl donor to a glycosyl sulfoxide (SPh → S(O)Ph) would enhance its reactivity enabling activation at lower temperatures.^12,13^ Hence, iodine control will perform better at lower temperatures restoring the kinetic control in challenging glycosylations as those using steroidal aglycones, favouring the selective formation of β-glycosides.

## Results and discussion

**Scheme 2:**
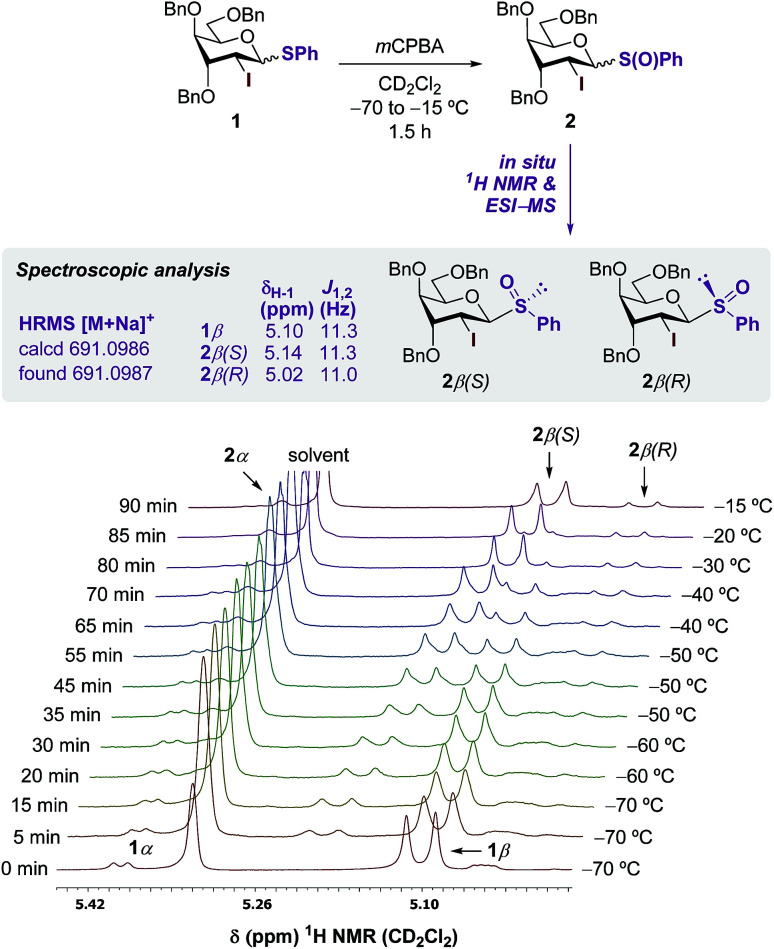
VT-NMR monitoring of the oxidation of 1.

Preliminary oxidation studies of 1 with *m*CPBA (in CHCl_3_)^13^ and Selectfluor™ (in CH_3_CN)^14^ revealed the high reactivity of the resulting sulfoxide 2, which evaded isolation due to decomposition. The best protocol used *m*CPBA as the sole oxidant in CH_2_Cl_2_ from −80 °C to −50 °C, followed by neutralization of the residual benzoic acid with NaHCO_3_, removal of the precipitate, and conducting the following glycosylation in sequence (Table 1). Thus, dichloromethane perfectly combines chemical inertness towards oxidants, good oxidation rate of sulfides using peroxy acids at the low temperatures necessitated to avoid decomposition,^15^ and good β-selective properties in the subsequent glycosylation reaction with sulfoxides (up to 3 : 1 β/α ratio with Bn as protecting groups).^13^ To verify the formation of 2, oxidation was monitored by ^1^H NMR in CD_2_Cl_2_ (Scheme 2). The signal peak at 5.10 ppm corresponding to the H-1 proton of the predominant 1β-anomer was gradually converted to two new doublets at 5.14 and 5.02 ppm, tentatively assigned to 2β(*S*) and 2β(*R*), respectively with a 88 : 12 dr. Although the signal of 2β(*S*) was gradually shifted upfield upon warming from −70 to −15 °C, the Δ*δ* of *ca.* 0.2 ppm between the two stereoisomers was in accordance with previously reported diastereomeric sulfoxides.^16^

**Table 1:** Glycosylation scope (SPh *vs.* S(O)Ph)^a^

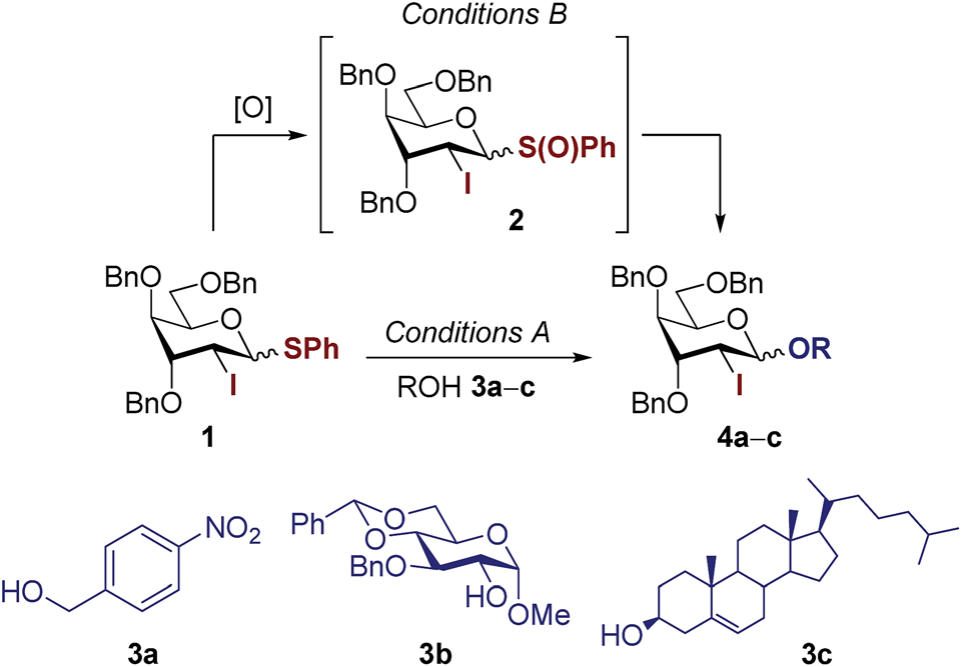				
Entry	ROH	Conditions	Yield^b^ (%)	β/α ratio^c^
1	3a	A	4a (72)	30 : 1
2	3a	B	4a (80)	40 : 1
3^d^	3b	A	4b (61)	16 : 1
4	3b	B	4b (69)	24 : 1
5^d^	3c	A	4c (66)	8 : 1
6	3c	B	4c (63)	21 : 1

a Conditions A: 1 (1 mmol), ROH 3a–c (2 mmol) and 4 Å molecular sieves (MS) in CH_2_Cl_2_ (4 mL) at −80 °C. Then, addition of NIS (3 mmol) and TfOH (0.2 mmol) at −80 °C to −40 °C. Conditions B: 1 (1 mmol), *m*CPBA (1.1 mmol) and 4 Å MS in CH_2_Cl_2_ (30 mL) at −80 °C. Then, NaHCO_3_ (5 mmol), filtration and addition of ROH 3a–c (2 mmol), DTBMP (3 mmol), 4 Å MS and Tf_2_O (2 mmol) at −80 °C.

b Isolated yield.

c Calculated by integration of anomeric protons in the ^1^H NMR spectrum of the crude reaction mixture.

d See ref. 8.

The identity of 2 was further confirmed by high-resolution mass spectrometry analysis (HRMS). Next, glycosylation was explored comparing the selectivities obtained for the activation of 1 and 2 (Table 1). Standard glycosylation using 1-thiophenyl donor 1 resulted in excellent β-stereoselectivities with primary 4-nitro-benzyl alcohol 3a (up to 30 : 1 β/α) and secondary methyl glucoside alcohol 3b (16 : 1 β/α) (entries 1 and 3).^8^ In contrast, employing cholesterol 3c as representative steroidal acceptor substantially decreased the selectivity to 8 : 1 β/α ratio and the thermodynamically more stable α-anomer could not be separated from its β-counterpart. Alternatively, oxidation of 1 followed by activation using the Tf_2_O/DTBMP system at −80 °C afforded the corresponding glycosides in very short reaction times and good yields (up to 80%). Glycosylation with primary benzylic 3a and secondary sugar acceptors 3b slightly improved the selectivity up to 40 : 1 β/α (entries 2 and 4). To our delight, glycosylations using cholesterol 3c reached comparable levels of stereocontrol (up to 21 : 1 β/α) only when sulfoxide was used as the glycosyl donor (entries 5 and 6). Thus, merging the excellent stereodirecting group properties of I^8,9^ and the lower reaction temperature enabled by the reactive sulfoxide ensured excellent kinetic control with challenging steroidal aglycones.

**Scheme 3:**
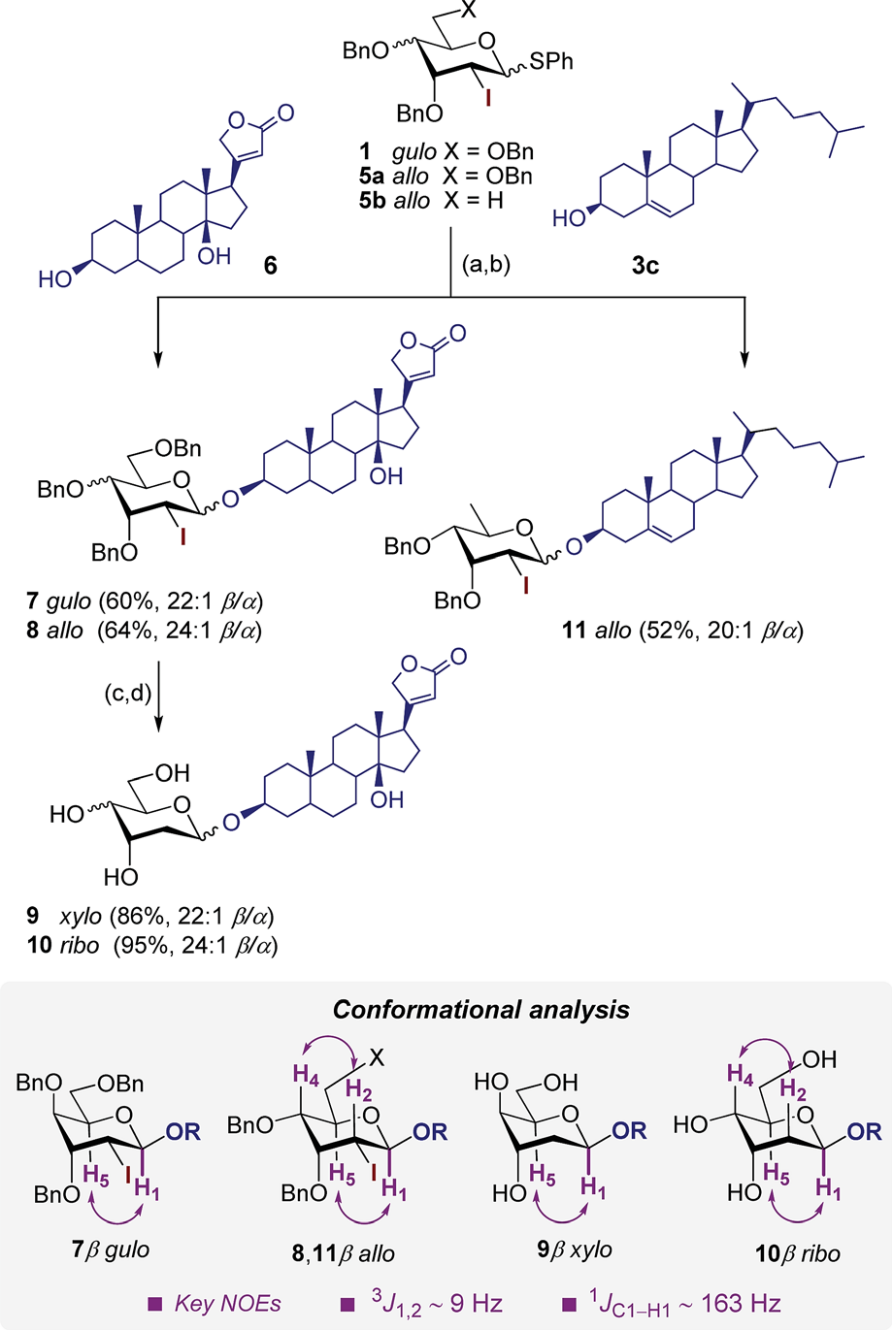
Synthesis of 2-deoxy- and 2,6-dideoxy-2-iodo-β-pyranosides 7, 8 and 11 and deprotection steps to digitoxigenyl 2-deoxy-β-d-*xylo* and d-*ribo* cardiac glycosides 9 and 10. Reagents and conditions: (a) *m*CPBA, 4 Å MS, CH_2_Cl_2_ from −80 °C to −40 °C, 30 min; (b) 3,6, DTBMP, 4 Å MS, Tf_2_O, −80 °C, 30 min; (c) Bu_3_SnH, Et_3_B, toluene, rt, 1 h; (d) H_2_ (1 atm), 10% Pd/C, 1 : 1 EtOAc/MeOH, 0 °C, 1–3 h.

Finally, the unique combination of oxidation/glycosylation sequence of this strategy was utilized for the synthesis of 2-deoxy-2-iodo-β-pyranosides 7 and 8 with high stereoselectivities (>22 : 1 β/α) and good yields (up to 64%) using the steroidal acceptor digitoxigenin 6 and d-*gulo*- and d-*allo*-1-thiopyranosides 1 and 5a as glycosyl donors (Scheme 3). The stereochemistry of the C-4 substituent had little effect on the selectivity although the slight improvement in the d-*allo* configuration may be explained by the less entropically disfavored β-transition state resulting from the stabilizing pseudoaxial positioning of OBn in the ^3^H_4_ conformer (Scheme 1).^11,17^ Elaboration of 7 and 8 under conventional deiodination and debenzylation conditions^2^ afforded final 2-deoxy cardiac glycosides 9 and 10 in excellent yields (up to 95%).

Likewise, cholesterol 3c was subjected to the same oxidation/glycosylation sequence with the more challenging 2,6-dideoxy glycosyl donor 5b to afford 2,6-dideoxy-2-iodo-d-*allo* derivative 11 with good stereoselectivity (20 : 1 β/α)^2,9*d*^ and moderate overall yield (52%). Final products 9 and 10 as well as their precursors 7, 8 and 11 adopted a ^4^C_1_ conformation as determined by NOE experiments and the analysis of diagnostic coupling constants (^3^*J*_1,2_ ∼ 9 Hz and ^1^*J*_C1–H1_ ∼ 163 Hz).

## Conclusions

In conclusion, the present work upgrades the previous reported methodology (using 1-thioglycosides) for the stereoselective synthesis of 2-deoxy-β-glycosides with d-*ribo* and d-*xylo* configurations, improving the overall β-control using challenging steroidal aglycones. The enhanced reactivity of glycosyl sulfoxides and the presence of an equatorial steering iodine permitted the precise formation of complex 2-deoxy-β-glycosides after removal of the temporary directing element. We expect that the present protocol will find broad application in the chemical synthesis of steroidal glycosides for the medicinal research field.

## Conflicts of interest

There are no conflicts to declare.

## Supplementary Material

RA-008-C8RA06619A-s001
