# Supplementary material for: Highly reactive 2-deoxy-2-iodo-d-allo and d-gulo pyranosyl sulfoxide donors ensure β-stereoselective glycosylations with steroidal aglycones
Source: RSC Adv. 2018 Aug 24;8(53):30076–9. doi: 10.1039/c8ra06619a (PMC9085402; doi:10.1039/c8ra06619a)
Supplement: RA-008-C8RA06619A-s001 [file RA-008-C8RA06619A-s001.pdf]

Electronic Supplementary Material (ESI) for RSC Advances.
This journal is © The Royal Society of Chemistry 2018

Electronic Supplementary Information

# Highly Reactive 2-Deoxy-2-iodo-D-allo and D-Gulopyranosyl Sulfoxide Donors Ensure *β*-Stereoselective Glycosylations with Steroidal Aglycones

Jordi Mestre, David Collado, David Benito-Alifonso, Miguel A. Rodríguez, M. Isabel Matheu, Yolanda Díaz, Sergio Castillón and Omar Boutureira*

*Departament de Química Analítica i Química Orgànica, Universitat Rovira i Virgili, C/Marcel·lí Domingo 1, 43007 Tarragona, Spain.*

**E-mail: omar.boutureira@urv.cat*

## Table of Contents

1. General Remarks S2
2. Experimental Section S2
3. References S10
4. NMR Spectra S11

## 1. General Remarks

Proton ($^1$H NMR) and carbon ($^{13}$C NMR) nuclear magnetic resonance spectra were recorded on a Varian Mercury spectrometer or a Bruker Avance Ultrashield (400 MHz for $^1$H) and (100.6 MHz for $^{13}$C). Spectra were fully assigned using COSY, HSQC, HMBC, and NOESY. All chemical shifts are quoted on the $\delta$ scale in ppm using the residual solvent as internal standard ($^1$H NMR: $CDCl_3$ = 7.26, $CD_3OD$ = 3.31 and $^{13}$C NMR: $CDCl_3$ = 77.16, $CD_3OD$ = 49.0). Coupling constants ($J$) are reported in Hz with the following splitting abbreviations: s = singlet, d = doublet, t = triplet, q = quartet, quin = quintet, and app = apparent. Infrared (IR) spectra were recorded on a Jasco FT/IR-600 Plus ATR Specac Golden Gate spectrophotometer. Absorption maxima ($\nu_{max}$) are reported in wavenumbers (cm$^{-1}$). High-resolution mass spectra (HRMS) were recorded on an Agilent 1100 Series LC/MSD mass spectrometer with electrospray ionization (ESI). Nominal and exact *m/z* values are reported in Daltons. Thin layer chromatography (TLC) was carried out using commercial aluminium backed sheets coated with $60F_{254}$ silica gel. Visualization of the silica plates was achieved using a UV lamp ($\lambda_{max}$ = 254 nm), 6% $H_2SO_4$ in EtOH. Flash column chromatography was carried out using silica gel 60 A CC (230–400 mesh). Mobile phases are reported in relative composition (*e.g.* 1:1 EtOAc/hexane v/v). HPLC grade dichloromethane ($CH_2Cl_2$) was dried using standard methods. All other solvents were used as supplied (Analytical or HPLC grade), without prior purification. All reagents were used as received from commercial suppliers. All reactions using anhydrous conditions were performed using flame-dried apparatus under an atmosphere of argon. Brine refers to a saturated solution of sodium chloride. Anhydrous sodium sulfate ($Na_2SO_4$) was used as drying agent after reaction work-up, as indicated.

## 2. Experimental Section

**General Method A for Glycosylation.** A mixture of donor **1**[1] (1 mmol), acceptor **3a–c** (2 mmol) and 4 Å MS in $CH_2Cl_2$ (4 mL) was stirred at –80 ºC for 30 min. NIS (3 mmol) and TfOH (0.2 mmol) were subsequently added and allowed to react at the indicated time and temperature. The reaction mixture was then diluted with EtOAc and washed with saturated aqueous $Na_2S_2O_3$ and $NaHCO_3$. The organic layer was dried with $Na_2SO_4$, filtered and the

solvent evaporated. The residue was then purified by flash column chromatography to afford **4a**–**c**.

**General Method B for Consecutive Oxidation and Glycosylation.** A mixture of donor **1**[1] (1 mmol) and 4 Å MS in $CH_2Cl_2$ (30 mL) was cooled to –80 ºC and stirred for 30 min. Then *m*CPBA (1.05 mmol) was added and the reaction mixture was stirred at –80ºC and the temperature was gradually warmed to –50 ºC until TLC showed completion of the reaction. $NaHCO_3$ (5 mmol) was added and the reaction mixture was stirred for 15 min at –80 ºC. The supernatant was transferred under argon to a Schlenk flask containing acceptor **3a**–**c** (2 mmol), DTBMP (3 mmol) and 4 Å MS at –80 ºC and the mixture stirred at this temperature for 30 min. $Tf_2O$ (2 mmol) was added and the reaction mixture was stirred at –80 ºC for 30 min. The reaction mixture was diluted with EtOAc, washed with saturated aqueous $NaHCO_3$, dried with anhydrous $Na_2SO_4$, filtered and the solvent evaporated. The residue was then purified by flash column chromatography to afford **4a**–**c**.

***p*-Nitrobenzyl 3,4,6-Tri-*O*-benzyl-2-deoxy-2-iodo-*α*/*β*-D-gulopyranoside (4a).** The title

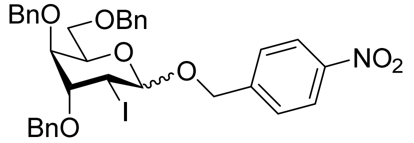


compound was prepared following the general method B above, starting from **1**[1] (29.3 mg, 0.045 mmol), 4 Å MS (70 mg), *m*CPBA (10.6 mg, 0.047 mmol) and $CH_2Cl_2$ (1.3 mL). After addition of $NaHCO_3$ (18.9 mg, 0.23 mmol) and filtration of the solution, the glycosylation was performed using **3a** (14 mg, 0.09 mmol), DTBMP (27.7mg, 0.135 mmol), $Tf_2O$ (15 *µ*L, 0.09 mmol) and 4 Å MS (20 mg) at –80 ºC for 30 min. After standard work-up, the residue was purified by flash column chromatography (from hexane to 1:3 EtOAc/hexane) to afford **4a** (25 mg, 80%) as an inseparable 40:1 *β*/*α* anomeric mixture as a faint yellow syrup. Data obtained from the mixture. $R_f$ (1:4 EtOAc/hexane): 0.33; Found: C, 58.81; H, 4.95; N, 1.98. $C_{34}H_{34}INO_7$ requires C, 58.71; H, 4.93; N, 2.01%; FTIR–ATR (neat, $\nu_{max}$): 2929, 2866, 1455, 1260, 1072, 1015, 800, 697; HRMS (TOF ES$^+$) *m*/*z*: $[M+Na]^+$ Calcd for $C_{34}H_{34}INNaO_7^+$ 718.1272; Found 718.1265. Data for **4a*β***: $^1H$ NMR ($CDCl_3$, 400 MHz) δ 8.18 (d, *J* = 8.6 Hz, 2H), 7.56 (d, *J* = 8.6 Hz, 2H), 7.40–7.17 (m, 15H), 4.96 (d, *J* = 13.2 Hz, 1H), 4.86 (d, *J* = 9.0 Hz, 1H), 4.67 (d, *J* = 13.2 Hz, 1H), 4.65 (d, *J* = 11.2 Hz, 1H), 4.54–4.37 (m, 6H), 4.20 (appt, *J* = 6.5 Hz, 1H), 3.81 (appt, *J* = 3.3 Hz,

1H), 3.61 (dd, *J* = 9.6, 6.4 Hz, 1H), 3.56 (dd, *J* = 9.6, 6.8 Hz, 1H), 3.38 (dd, *J* = 3.6, 1.2 Hz, 1H); $^{13}$C NMR ($CDCl_3$, 100.6 MHz) δ 147.4, 145.1, 138.1, 137.5, 137.4, 128.6, 128.5, 128.4, 128.3, 128.0, 127.9, 127.8, 123.6, 99.8, 78.6, 74.1, 73.7, 73.6, 73.1, 73.0, 69.8, 68.9, 31.4.

**Methyl (3',4',6'-Tri-*O*-benzyl-2'-deoxy-2'-iodo-*α*/*β*-D-gulopyranosyl)-(1→2)-3-*O*-benzyl-4,6-*O*-benzylidene-*α*-D-glucopyranoside (4b).**[1] The title compound was prepared following the general method B above, starting from **1**[1] (19.3 mg, 0.03 mmol), 4 Å MS (40 mg), *m*CPBA (7.1 mg, 0.031 mmol) and $CH_2Cl_2$ (0.9 mL). After addition of $NaHCO_3$ (12.6 mg, 0.15 mmol) and filtration of the solution, the glycosylation was performed using **3b** (9.3 mg, 0.06 mmol), DTBMP (18.5 mg, 0.09 mmol), $Tf_2O$ (10 *μ*L, 0.06 mmol) and 4 Å MS (13 mg). After stirring for 30 min at –80 ºC, standard work-up was performed and the residue was purified by flash column chromatography (from hexane to 1:3 EtOAc/hexane) to afford **4b** (19 mg, 69%) as a 24:1 *β*/*α* anomeric mixture as a colorless syrup. Data obtained from the mixture. $R_f$ (1:3 EtOAc/hexane): 0.31; FTIR–ATR (neat, $\nu_{max}$): 2921, 2861, 1454, 1371, 1072, 994, 740, 697; HRMS (TOF ES$^+$) *m*/*z*: [M+Na]$^+$ Calcd for $C_{48}H_{51}INaO_{10}^+$ 937.2419; Found 937.2412. Data for **4b***β*: $^1$H NMR ($CDCl_3$, 400 MHz) δ 7.50–7.12 (m, 25H), 5.54 (s, 1H), 5.08 (d, *J* = 9.2 Hz, 1H), 5.07 (d, *J* = 10.6 Hz, 1H), 4.86 (d, *J* = 3.7 Hz, 1H), 4.80 (d, *J* = 10.5 Hz, 1H), 4.66 (d, *J* = 11.5 Hz, 1H), 4.50–4.38 (m, 6H), 4.28 (dd, *J* = 10.1, 4.8 Hz, 1H), 4.16 (td, *J* = 6.4, 1.2 Hz, 1H), 4.03 (t, *J* = 9.3 Hz, 1H), 3.85 (td, *J* = 10.0, 4.8 Hz, 1H), 3.80 (t, *J* = 3.2 Hz, 1H), 3.77–3.69 (m, 2H), 3.59 (t, *J* = 9.4 Hz, 1H), 3.53 (d, *J* = 6.5 Hz, 2H), 3.39 (s, 3H), 3.36 (dd, *J* = 3.4, 1.4 Hz, 1H); $^{13}$C NMR ($CDCl_3$, 100.6 MHz) δ 138.8, 138.1, 137.7, 137.6, 129.0, 128.6, 128.5, 128.3, 128.2, 128.1, 127.8, 127.5, 126.1, 101.4, 100.4, 82.8, 79.2, 79.1, 77.7, 75.3, 74.1, 73.9, 73.5, 73.0, 72.8, 69.3, 69.0, 62.2, 55.4, 30.7.

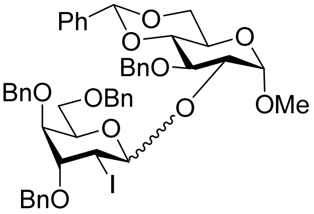


**Cholesteryl 3,4,6-Tri-*O*-benzyl-2-deoxy-2-iodo-*α*/*β*-D-gulopyranoside (4c).**[1] The title compound was prepared following the general method B above, starting from **1**[1] (10.3 mg, 0.016 mmol), 4 Å MS (20 mg), *m*CPBA (3.8 mg,

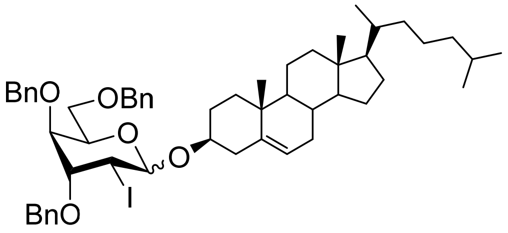

0.016 mmol) and $CH_2Cl_2$ (0.48 mL). After addition of $NaHCO_3$ (6.7 mg, 0.08 mmol) and filtration of the solution, the glycosylation was performed using **3c** (4.9 mg, 0.03 mmol), DTBMP (9.9 mg, 0.048 mmol), $Tf_2O$ (5.3 $\mu$L, 0.032 mmol) and 4 Å MS (6.9 mg). After stirring for 30 min at –80 ºC, standard work-up was performed and the residue was purified by flash column chromatography (from hexane to 1:3 EtOAc/hexane 1:3) to afford **4c** (9 mg, 63%) as a 21:1 $\beta/\alpha$ anomeric mixture as a colorless syrup. Data obtained from the mixture. $R_f$ (1:3 EtOAc/hexane): 0.62; FTIR–ATR (neat, $\nu_{max}$): 2929, 2865, 1455, 1260, 1072, 1015, 800, 697; HRMS (TOF ES$^+$) *m/z*: [M+Na]$^+$ Calcd for $C_{54}H_{73}INaO_5^+$ 951.4395; Found 951.4391. Data for **4c**$\beta$: $^1$H NMR ($CDCl_3$, 400 MHz) δ 7.39–7.13 (m, 15H), 5.35 (bs, 1H), 4.82 (d, *J* = 9.5 Hz, 1H), 4.64 (d, *J* = 11.4 Hz, 1H), 4.53–4.32 (m, 6H), 4.16 (t, *J* = 6.3 Hz, 1H), 3.78 (t, *J* = 3.2 Hz, 1H), 3.56 (d, *J* = 6.4 Hz, 2H), 3.49 (m, 1H), 3.34 (d, *J* = 3.5 Hz, 1H ), 2.39–0.67 (m, 44H); $^{13}$C NMR ($CDCl_3$, 100.6 MHz) δ 140.9, 137.8, 137.6, 128.6, 128.5, 128.4, 128.3, 128.2, 128.1, 127.8, 121.9, 98.9, 79.6, 78.9, 74.0, 73.7, 73.5, 72.9, 72.8, 69.1, 56.9, 56.3, 50.3, 42.5, 39.9, 39.7, 38.7, 37.4, 36.9, 36.3, 35.9, 33.5, 32.1, 32.0, 29.9, 29.7, 28.4, 28.2, 24.4, 24.0, 23.0, 22.7, 21.2, 19.6, 18.9, 12.0.

**Synthesis of phenyl 3,4-di-*O*-benzyl-2,6-dideoxy-2-iodo-1-thio-*α/β*-D-allopyranoside (5b).**

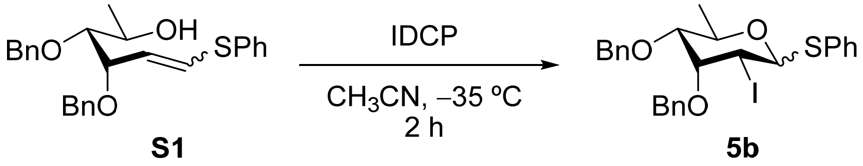


To a solution of **S1**[2] (2:5 *Z/E* ratio) (100 mg, mmol) in dry $CH_3CN$ (4 mL) at –35 °C was added IDCP (245 mg, 0.52 mmol). The mixture was left to stir at this temperature for 2 h. The reaction was diluted with $CH_2Cl_2$ and washed with saturated aqueous $Na_2S_2O_3$. The combined organic layers were dried over $MgSO_4$, filtered, and concentrated. The crude was purified by radial chromatography (from hexane to 1:3 EtOAc/hexane) to afford **5b** (51 mg, 40%) as an inseparable 1:1.6 $\alpha/\beta$ mixture as a yellowish syrup. Data obtained from the mixture. $R_f$ (1:3 EtOAc/hexane): 0.48. Data for **5b**$\alpha$: $^1$H NMR (400 MHz, $CDCl_3$) $\delta$ 7.57–

7.24 (m, 15H), 5.46 (s, 1H), 4.92–4.31 (m, 6H), 4.00 (dd, $J$ = 3.6, 2.8 Hz, 1H), 3.97 (d, $J$ = 2.8 Hz, 1H), 1.36 (d, $J$ = 6.4 Hz, 3H); $^{13}$C NMR (100.6 MHz, $CDCl_3$) $\delta$ 137.7, 137.3, 132.1, 130.9, 128.8, 128.5, 128.4, 128.1, 127.9, 127.6, 127.2, 89.3, 76.2, 75.6, 72.0, 71.4, 65.5, 27.3, 17.8. Data for **5b***β*: $^{1}$H NMR (400 MHz, $CDCl_3$) $\delta$ 7.57–7.22 (m, 15H), 5.10 (d, $J$ = 11.2 Hz, 1H), 4.90–4.42 (m, 2H), 4.18 (dd, $J$ = 2.6, 2.2 Hz, 1H), 4.11 (dq, $J$ = 9.6, 6.4 Hz, 1H), 4.03 (dd, $J$ = 11.2, 2.6 Hz, 1H), 3.23 (dd, $J$ = 9.6, 2.2 Hz, 1H), 1.27 (d, $J$ = 6.4 Hz, 3H); $^{13}$C NMR (100.6 MHz, $CDCl_3$) $\delta$ 138.1, 137.4, 132.9, 131.5, 128.9, 128.7, 128.5, 128.2, 128.0, 127.8, 127.3, 84.4, 81.6, 78.3, 75.7, 72.3, 72.2, 32.2, 18.2.

**Digitoxigenyl 3,4,6-Tri-*O*-benzyl-2-deoxy-2-iodo-*α*/*β*-D-gulopyranoside (7).** The title compound was prepared following the general method B above, starting from **1**[1] (14.1 mg, 0.022 mmol), 4 Å MS (25 mg), *m*CPBA (5.2 mg, 0.022 mmol) and $CH_2Cl_2$ (0.7 mL). After addition of $NaHCO_3$ (9.2 mg, 0.11 mmol) and filtration of the solution, the glycosylation was performed using **6** (6.7 mg, 0.04 mmol), DTBMP (13.6 mg, 0.066 mmol), $Tf_2O$ (7.3 $\mu$L, 0.044 mmol) and 4 Å MS (9 mg). After stirring at –80 ºC for 30 min, standard work-up was performed and the residue was purified by flash column chromatography (from hexane to 1:2 EtOAc/hexane) to afford **7** (12 mg, 60%) as a 22:1 *β*/*α* anomeric mixture as a colorless syrup. Data obtained from the mixture. $R_f$ (1:1 EtOAc/hexane): 0.44; FTIR–ATR (neat, $\nu_{max}$): 3488, 2923, 2854, 1742, 1454, 1067, 1025, 736, 698; HRMS (TOF ES$^+$) *m*/*z*: [M+Na]$^+$ Calcd for $C_{50}H_{61}INaO_8^+$ 939.3303; Found 939.3292. Data for **7***β*: $^{1}$H NMR (400 MHz, $CDCl_3$) δ 7.41–7.14 (m, 15H), 5.88 (bs, 1H), 5.00 (dd, $J$ = 18.2, 1.5 Hz, 1H), 4.82 (dd, $J$ = 18.3, 1.5 Hz, 1H), 4.77 (d, $J$ = 9.2 Hz, 1H), 4.66 (d, $J$ = 11.6 Hz, 1H), 4.53–4.34 (m, 6H), 4.15 (t, $J$ = 6.3 Hz, 1H), 3.99 (bs, 1H), 3.79 (t, $J$ = 3.3 Hz, 1H), 3.59–3.50 (m, 2H), 3.34 (dd, $J$ = 3.2, 0.8 Hz, 1H), 2.79 (m, 1H), 2.20–1.18 (m, 21H), 0.94 (s, 3H), 0.87 (s, 3H); $^{13}$C NMR (100.6 MHz, $CDCl_3$) δ 174.7, 138.3, 137.7, 137.6, 128.6, 128.5, 128.4, 128.2, 127.8, 127.7, 117.8, 98.1, 85.8, 78.9, 74.1, 73.9,

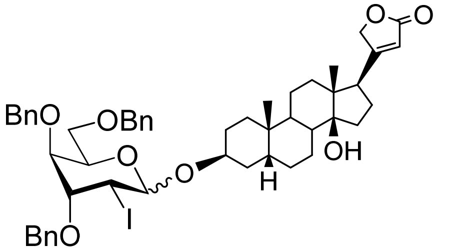

73.8, 73.6, 73.5, 72.9, 72.8, 69.1, 51.0, 49.7, 42.0, 40.2, 36.1, 36.0, 35.2, 33.4, 33.3, 30.1, 29.8, 29.3, 27.0, 26.5, 23.7, 21.5, 21.3, 15.9.

**Digitoxigenyl 3,4,6-Tri-*O*-benzyl-2-deoxy-2-iodo-*α*/*β*-D-allopyranoside (8).** The title compound was prepared following the general method B above, starting from **5a**[1] (15.4 mg, 0.024 mmol), 4 Å MS (25 mg), *m*CPBA (5.7 mg, 0.024 mmol) and $CH_2Cl_2$ (0.8 mL). After addition of $NaHCO_3$ (10 mg, 0.12 mmol) and filtration of the solution, the glycosylation was performed using **6** (7.3 mg, 0.044 mmol), DTBMP (14.8 mg, 0.072 mmol), $Tf_2O$ (7.9 $\mu$L, 0.048 mmol) and 4 Å MS (10 mg). After stirring at –80 ºC for 30 min, standard work-up was performed and residue was purified by flash column chromatography (from hexane to 1:2 EtOAc/hexane) to afford **8** (14 mg, 64%) as a 24:1 *β*/*α* anomeric mixture as a colorless syrup. Data obtained from the mixture. $R_f$ (1:1 EtOAc/hexane): 0.45; FTIR–ATR (neat, $\nu_{max}$): 3490, 2924, 2854, 1742, 1454, 1067, 1026, 737, 698; HRMS (TOF $ES^+$) *m*/*z*: $[M+Na]^+$ Calcd for $C_{50}H_{61}INaO_8^+$ 939.3303; Found 939.3397. Data for **8***β*: $^1$H NMR (400 MHz, $CDCl_3$) δ 7.49–7.23 (m, 15H), 5.87 (bs, 1H), 4.99 (d, *J* = 17.9 Hz, 1H), 4.88 (d, *J* = 10.5 Hz, 1H), 4.80 (d, *J* = 17.9 Hz, 1H), 4.79 (d, *J* = 8.0 Hz, 1H), 4.78 (d, *J* = 10.4 Hz, 1H), 4.67–4.48 (m, 4H), 4.17 (bs, 1H), 4.12 (m, 1H), 4.05 (dd, *J* = 9.0, 2.4 Hz, 1H), 3.96 (bs, 1H), 3.75–3.67 (m, 2H), 3.64 (dd, *J* = 10.9, 4.7 Hz, 1H), 2.78 (m, 1H), 2.23–1.14 (m, 21H), 0.94 (s, 3H), 0.87 (s, 3H); $^{13}$C NMR (100.6 MHz, $CDCl_3$) δ 174.7, 138.5, 137.7, 128.6, 128.5, 128.3, 128.2, 128.1, 128.0, 127.8, 127.7, 117.8, 98.7, 85.8, 78.6, 75.8, 74.5, 73.6, 73.5, 73.2, 72.3, 69.4, 51.1, 49.7, 42.0, 40.2, 36.2, 36.0, 35.3, 33.3, 30.2, 29.9, 29.6, 27.0, 26.6, 26.5, 23.8, 21.5, 21.3, 15.9.

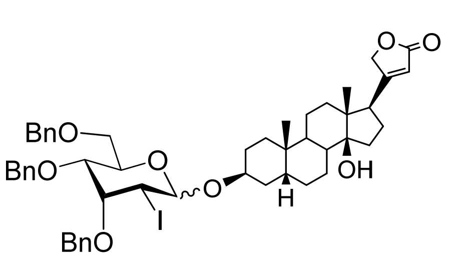


**Digitoxigenyl 2-Deoxy-*α*/*β*-D-*xylo*-pyranoside (9).** To a solution of **7** (16 mg, 0.017 mmol) in toluene (0.17 mL) were consecutively added $Bu_3SnH$ (27 mL, 0.10 mmol) and $Et_3B$ (10 mL, 0.01 mmol). The reaction mixture was stirred at room temperature for 1 h, then diluted with EtOAc and washed with saturated aqueous $NaHCO_3$. The combined

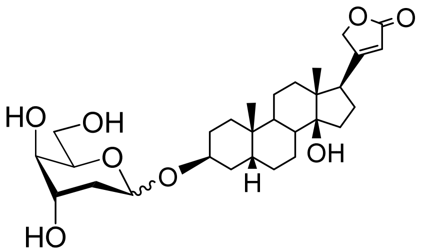

organic extracts were dried over anhydrous $Na_2SO_4$ and concentrated under reduced pressure. The crude was filtered through a short path of $SiO_2$ (from 1:9 to 1:1 EtOAc/hexane and 5% $Et_3N$) to remove tin contaminants. Fractions containing the crude product were concentrated under reduced pressure, dissolved in 1:1 EtOAc/MeOH (0.7 mL) and 10% Pd/C (19 mg) was added. The mixture was stirred at 0 ºC under $H_2$ atmosphere (1 atm). After 1 h, the reaction mixture was diluted with EtOAc and filtered through a short path of Celite®. The residue was purified by flash column chromatography (from EtOAc to 95:5 EtOAc/MeOH and 5% $Et_3N$) to afford **9** (7.6 mg, 86% over two steps) as a 22:1 *β*/*α* anomeric mixture as a colorless syrup. Data obtained from the mixture. $R_f$ (1:9 MeOH/$CH_2Cl_2$): 0.33; FTIR–ATR (neat, $\nu_{max}$): 3392, 2923, 2853, 1754, 1456, 1024; HRMS (TOF $ES^+$) *m*/*z*: $[M+Na]^+$ Calcd for $C_{29}H_{44}NaO_8^+$ 543.2928; Found 543.2918. Data for **9***β*: $^1H$ NMR (400 MHz, $CD_3OD$) δ 5.84 (bs, 1H), 5.04 (d, $J$ = 18.6 Hz, 1H), 4.90–4.82 (m, 2H), 4.07 (bs, 1H), 3.95 (q, $J$ = 3.2 Hz, 1H), 3.84 (t, $J$ = 5.8 Hz, 1H), 3.76–3.71 (m, 2H), 3.47 (m, 1H), 2.80 (m, 1H), 2.21–2.04 (m, 2H), 2.03–1.13 (m, 21H), 0.91 (s, 3H), 0.85 (s, 3H); $^{13}C$ NMR (100.6 MHz, $CD_3OD$) δ 177.3, 176.6, 117.4, 96.8, 85.7, 74.6, 73.8, 73.4, 69.2, 68.6, 62.7, 51.6, 50.4, 41.9, 40.5, 37.0, 36.2, 35.7, 34.8, 33.0, 30.7, 30.3, 27.4, 27.2, 27.0, 24.0, 21.9, 21.7, 16.2.

**Digitoxigenyl 2-deoxy-*α*/*β*-D-*ribo*-pyranoside (10).** To a solution of **8** (9.8 mg, 0.0107 mmol) in toluene (0.4 mL) were consecutively added $Bu_3SnH$ (17.2 $\mu$L, 0.064 mmol) and $Et_3B$ (1 M, 6.4 $\mu$L, 0.006 mmol). The reaction mixture was stirred at room temperature for 1 h, then diluted with EtOAc and washed with saturated aqueous $NaHCO_3$. The combined organic extracts were dried with anhydrous $Na_2SO_4$ and concentrated under reduced pressure. The crude was filtered through a short path of $SiO_2$ (from 1:9 to 1:1 EtOAc/hexane and 5% $Et_3N$) to remove tin contaminants. Fractions containing the crude product were concentrated under reduced pressure, dissolved in 1:1 EtOAc/MeOH (0.7 mL) and 10% Pd/C (16 mg) was added. The mixture was stirred at 0 ºC under $H_2$ atmosphere (1 atm). After 3 h, the reaction mixture was diluted with EtOAc and filtered through a short path of Celite®. The residue was purified by flash column chromatography

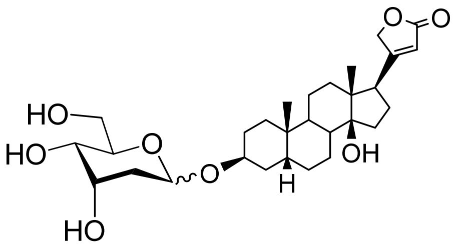

(from EtOAc to EtOAc/MeOH 95:5 and 5% $Et_3N$) to afford **10** (5.3 mg, 95% over two steps) as a 24:1 *β*/*α* anomeric mixture as a colorless syrup. Data obtained from the mixture. $R_f$ (1:9 MeOH/$CH_2Cl_2$): 0.29; FTIR–ATR (neat, $\nu_{max}$): 3392, 2924, 2854, 1736, 1450, 1025; HRMS (TOF ES$^+$) *m*/*z*: $[M+Na]^+$ Calcd for $C_{29}H_{44}NaO_8^+$ 543.2928; Found 543.2920. Data for **10***β*: $^1H$ NMR (400 MHz, $CD_3OD$) δ 5.90 (bt, *J* = 1.5 Hz, 1H), 5.04 (dd, *J* = 18.4, 1.6 Hz, 1H), 4.98–4.87 (m, 2H), 4.10 (m, 1H), 4.05 (q, *J* = 3.1 Hz, 1H), 3.82 (dd, *J* = 10.9, 1.8 Hz, 1H), 3.73–3.63 (m, 2H), 3.45 (dd, *J* = 9.4, 3.2 Hz, 1H), 2.83 (m, 1H), 2.24–2.12 (m, 2H), 1.98–1.22 (m, 21H), 0.95 (s, 3H), 0.88 (s, 3H); $^{13}C$ NMR (100.6 MHz, $CD_3OD$) δ 178.5, 177.3, 117.8, 96.9, 86.5, 75.5, 75.4, 74.1, 69.3, 69.1, 63.6, 52.1, 51.1, 42.7, 41.0, 39.5, 38.0, 36.9, 36.4, 34.1, 33.4, 31.4, 30.8, 28.1, 27.9, 27.6, 24.3, 22.6, 22.4.

**Cholesteryl 3,4-di-*O*-benzyl-2,6-dideoxy-2-iodo-1-thio-*α*/*β*-D-allopyranoside (11).**[2]

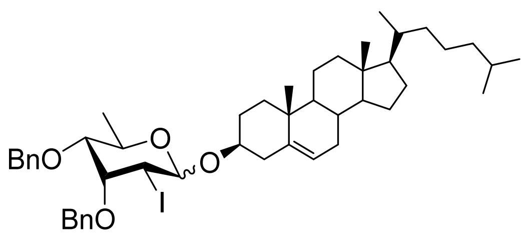


The title compound was prepared following the general method B above, starting from **5b** (10.1 mg, 0.018 mmol), 4 Å MS (20 mg), *m*CPBA (4.3 mg, 0.018 mmol) and $CH_2Cl_2$ (0.5 mL). After addition of $NaHCO_3$ (7.5 mg, 0.09 mmol) and filtration of the solution, the glycosylation was performed using **3c** (5 mg, 0.03 mmol), DTBMP (11.1 mg, 0.054 mmol), $Tf_2O$ (6 *μ*L, 0.036 mmol) and 4 Å MS (7 mg). After stirring at –80 ºC for 30 min, standard work-up was performed and the residue was purified by flash column chromatography (from hexane to 1:3 EtOAc/hexane) to afford **7c** (7.9 mg, 52%) as a 20:1 *β*/*α* anomeric mixture as a yellowish syrup. Data obtained from the mixture. Data for **11***β*: $^1H$ NMR (400 MHz, $CDCl_3$) *δ* 7.57–7.25 (m, 10H), 5.35 (d, *J* = 5.2 Hz, 1H), 4.90 (d, *J* = 10.5 Hz, 1H), 4.86 (d, *J* = 9.0 1H), 4.79 (d, *J* = 10.5 Hz, 1H), 4.68 (d, *J* = 11.6 Hz, 1H), 4.53 (d, *J* = 11.6 Hz, 1H), 4.16–3.98 (m, 3H), 3.51–3.40 (m, 1H), 3.30 (dd, *J* = 2.1, 9.3 Hz, 1H), 2.46–0.67 (m, 44H); $^{13}C$ NMR (100.6 MHz, $CDCl_3$) *δ* 140.7, 138.4, 137.6, 128.5, 128.14, 128.06, 128.0, 127.9,

127.6, 121.8, 98.8, 81.9, 79.7, 78.2, 75.6, 72.3, 69.1, 56.8, 56.2, 50.2, 42.4, 39.8, 39.5, 38.6, 37.3, 36.7, 36.2, 35.8, 33.6, 32.0, 31.9, 29.7, 29.5, 28.2, 28.0, 24.3, 23.8, 22.8, 22.6, 21.1, 19.4, 18.7, 18.1, 14.1, 11.9.

## 3. References


[1] Rodríguez, M. A.; Boutureira, O.; Arnés, X.; Díaz, Y.; Matheu, M. I.; Castillón, S. *J. Org. Chem.* **2005**, *70*, 10297–10310.

[2] Rodríguez, M. A.; Boutureira, O.; Matheu, M. I.; Díaz Y.; Castillón, S. *Eur. J. Org. Chem.* **2007**, 2470–10310.

**4. NMR Spectra**

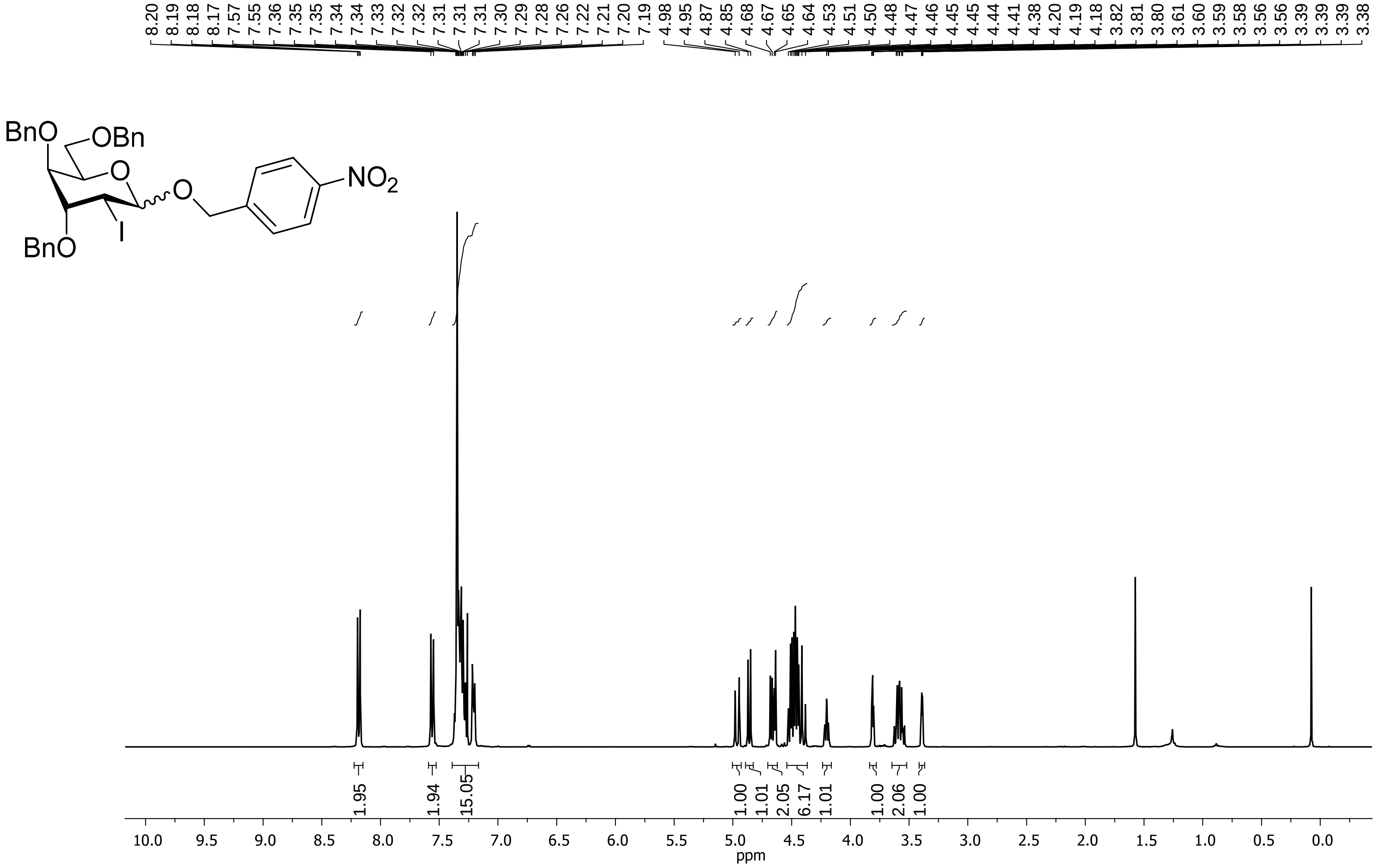


**Figure S1.** $^{1}$H NMR ($CDCl_3$, 400 MHz) of **4a**

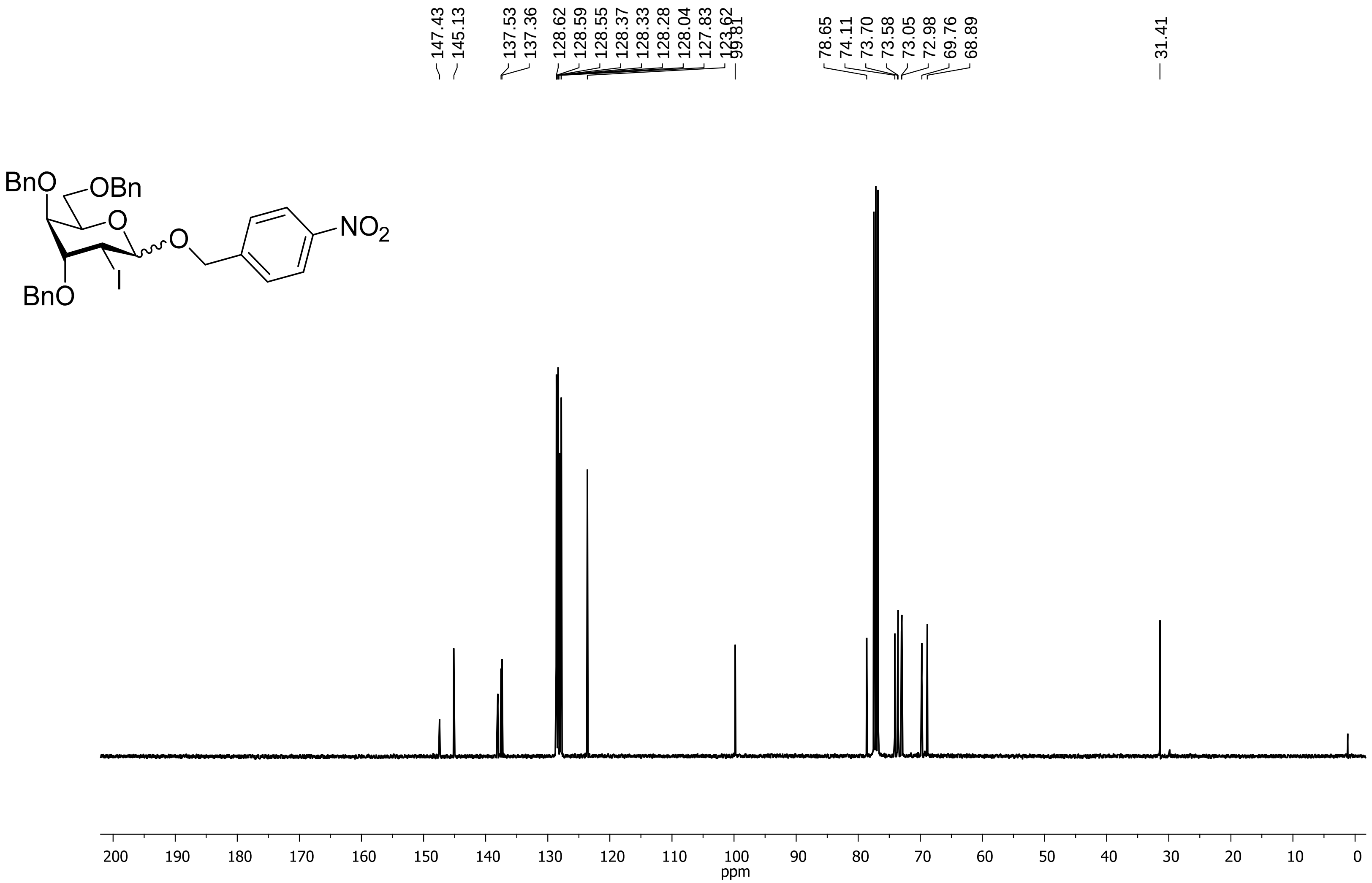


**Figure S2.** $^{13}$C NMR ($CDCl_3$, 100.6 MHz) of **4a**

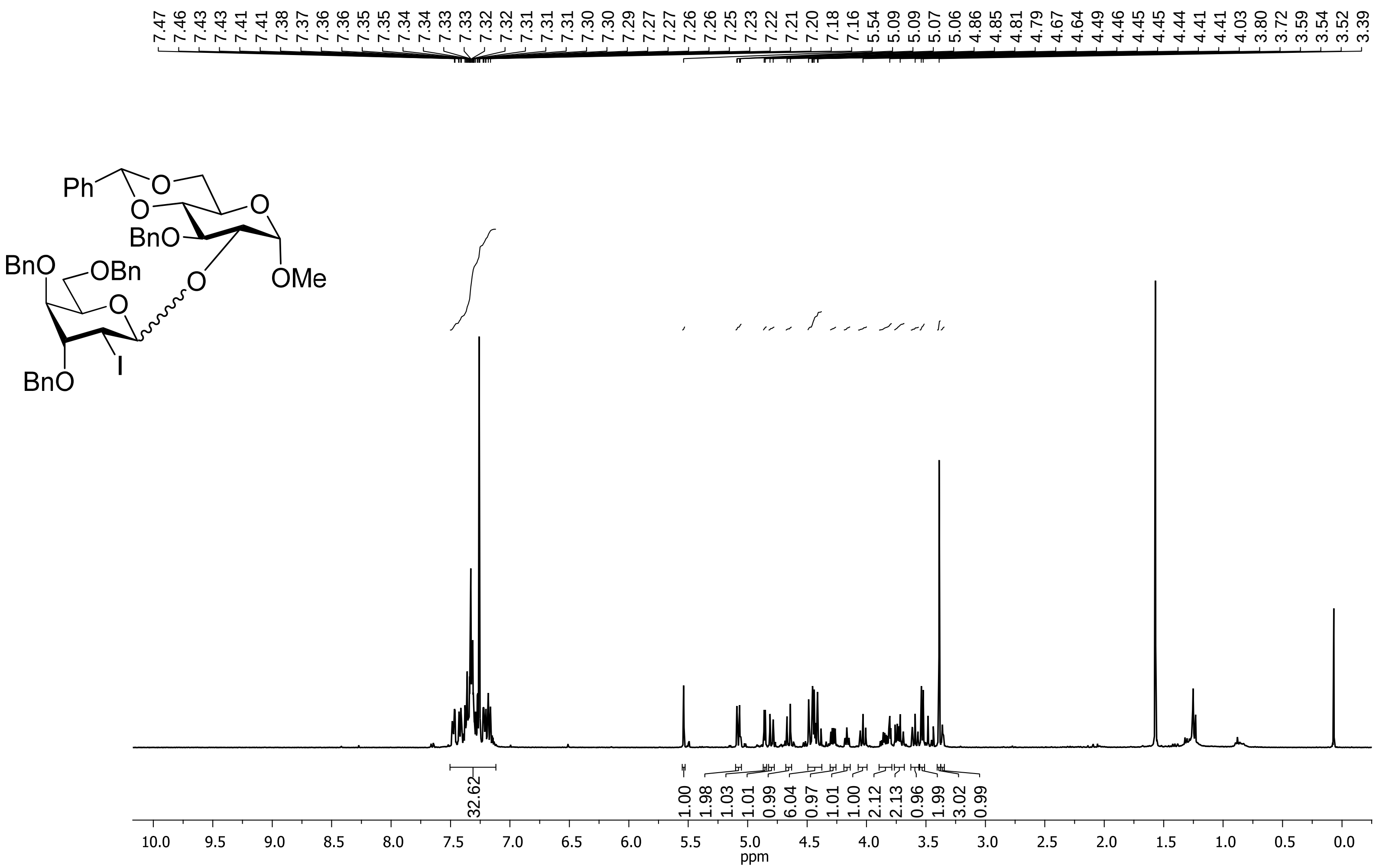


**Figure S3.** $^{1}$H NMR ($CDCl_3$, 400 MHz) of **4b**

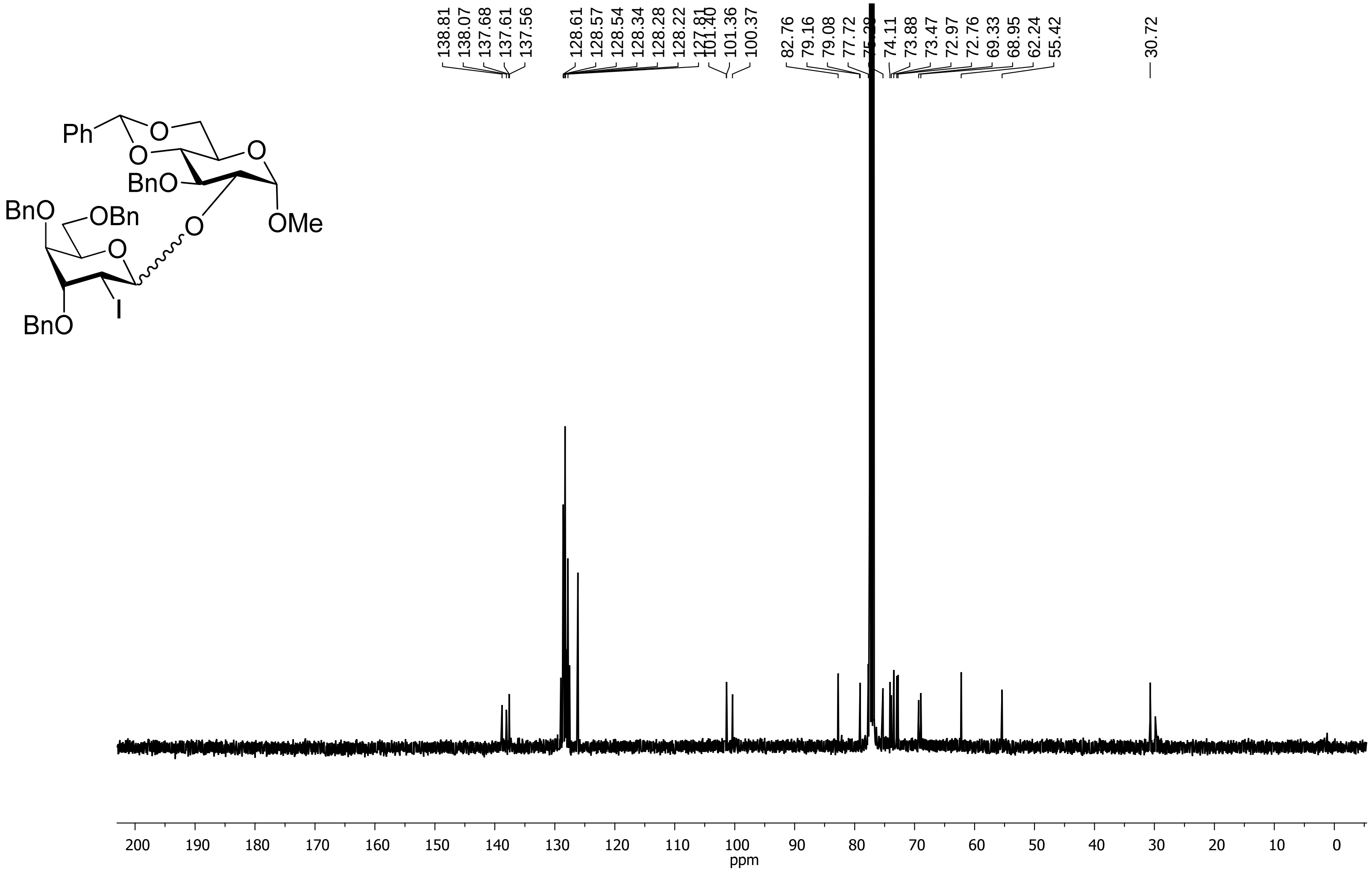


**Figure S4.** $^{13}$C NMR ($CDCl_3$, 100.6 MHz) of **4b**

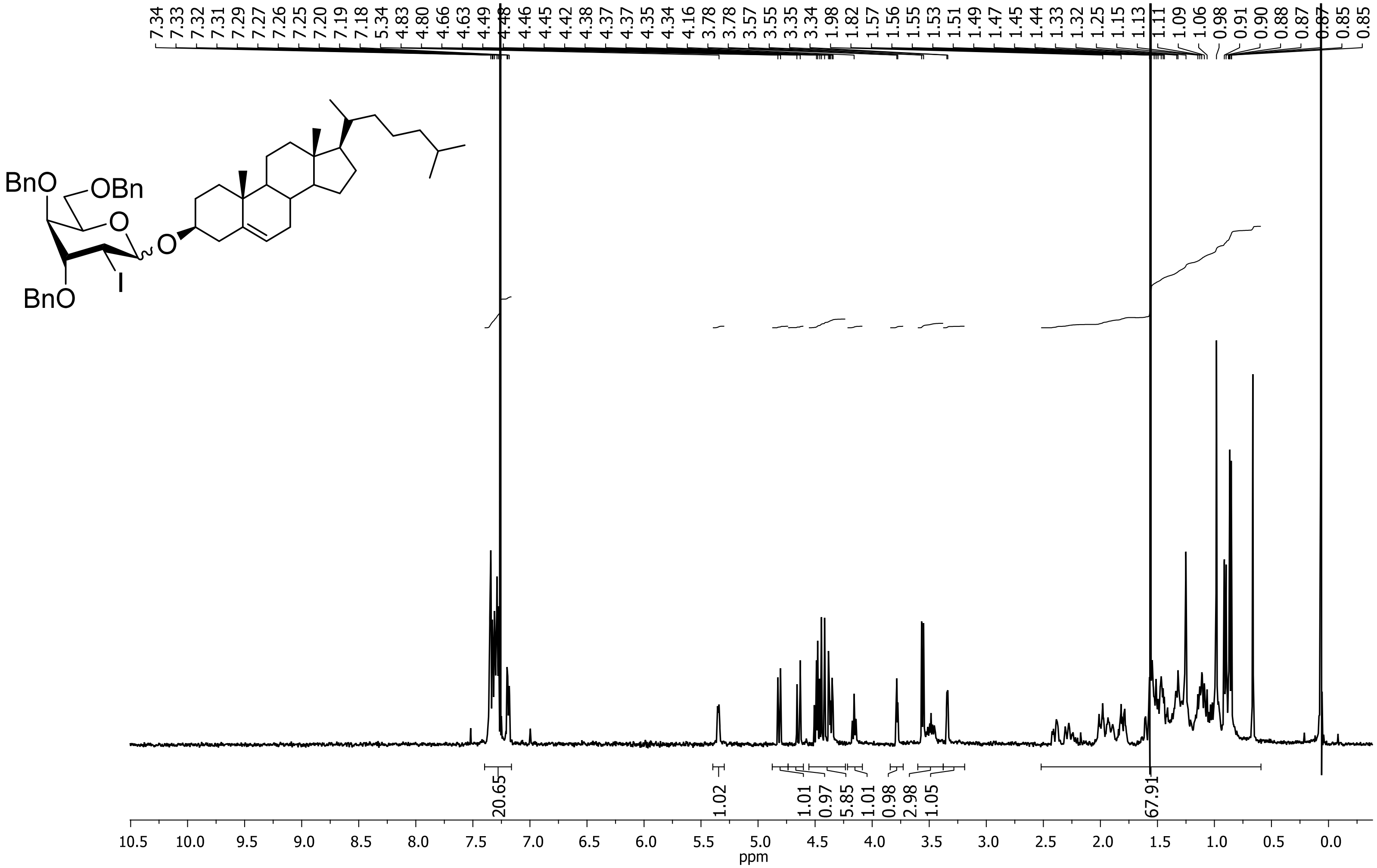


**Figure S5.** $^{1}$H NMR ($CDCl_3$, 400 MHz) of **4c**

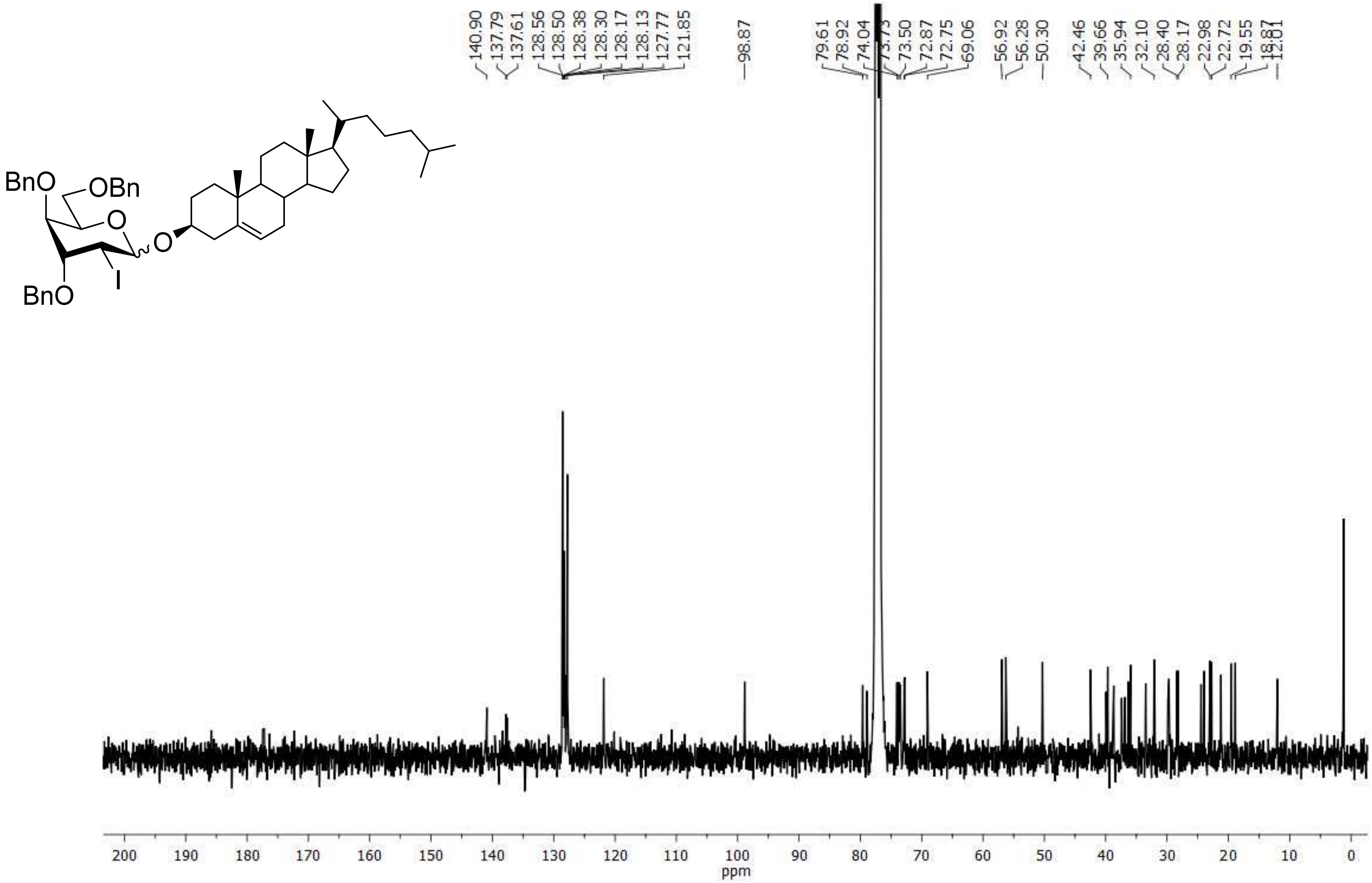


**Figure S6.** $^{13}$C NMR ($CDCl_3$, 100.6 MHz) of **4c**

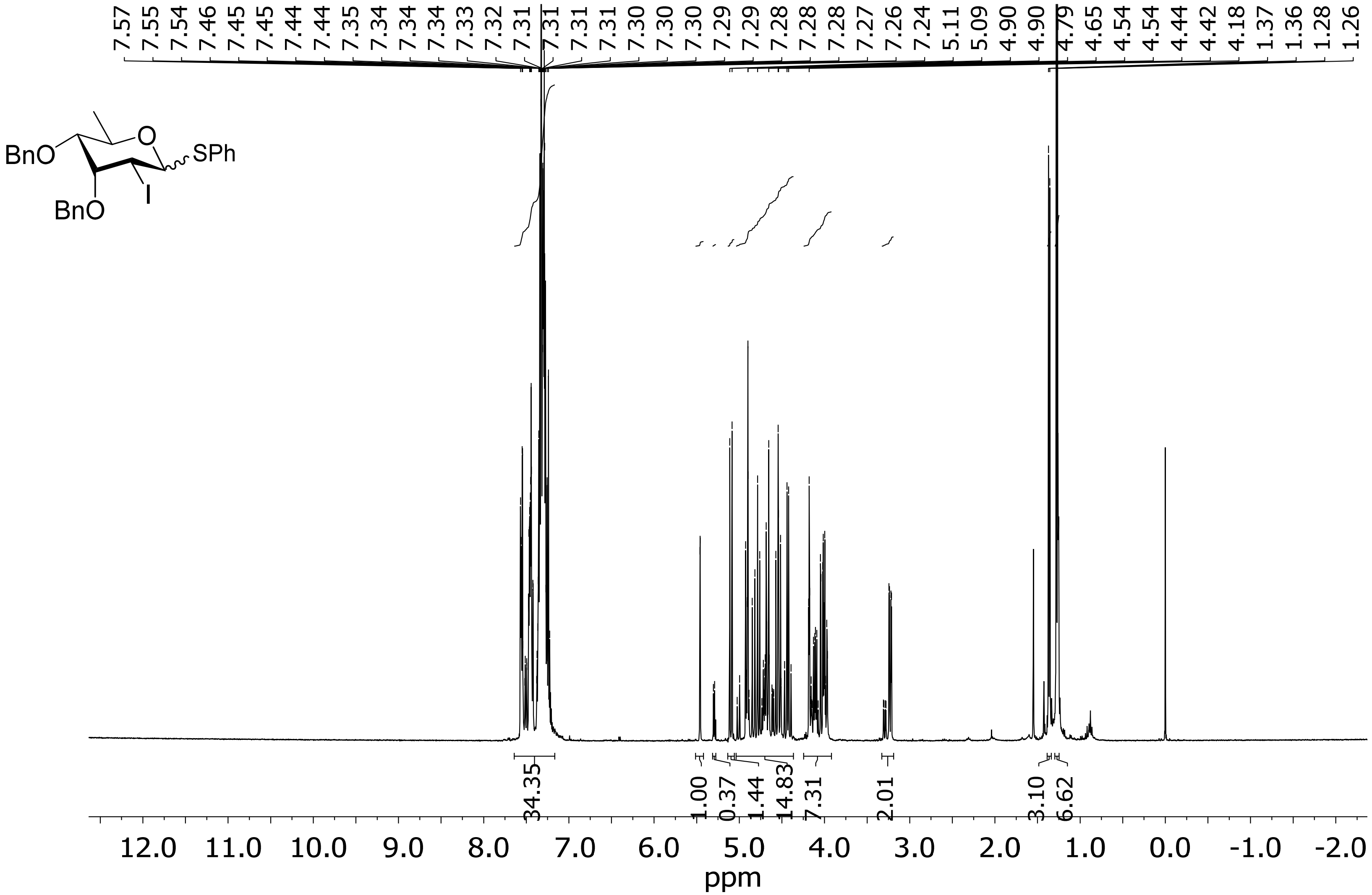


**Figure S7.** $^1$H NMR ($CDCl_3$, 400 MHz) of **5b**

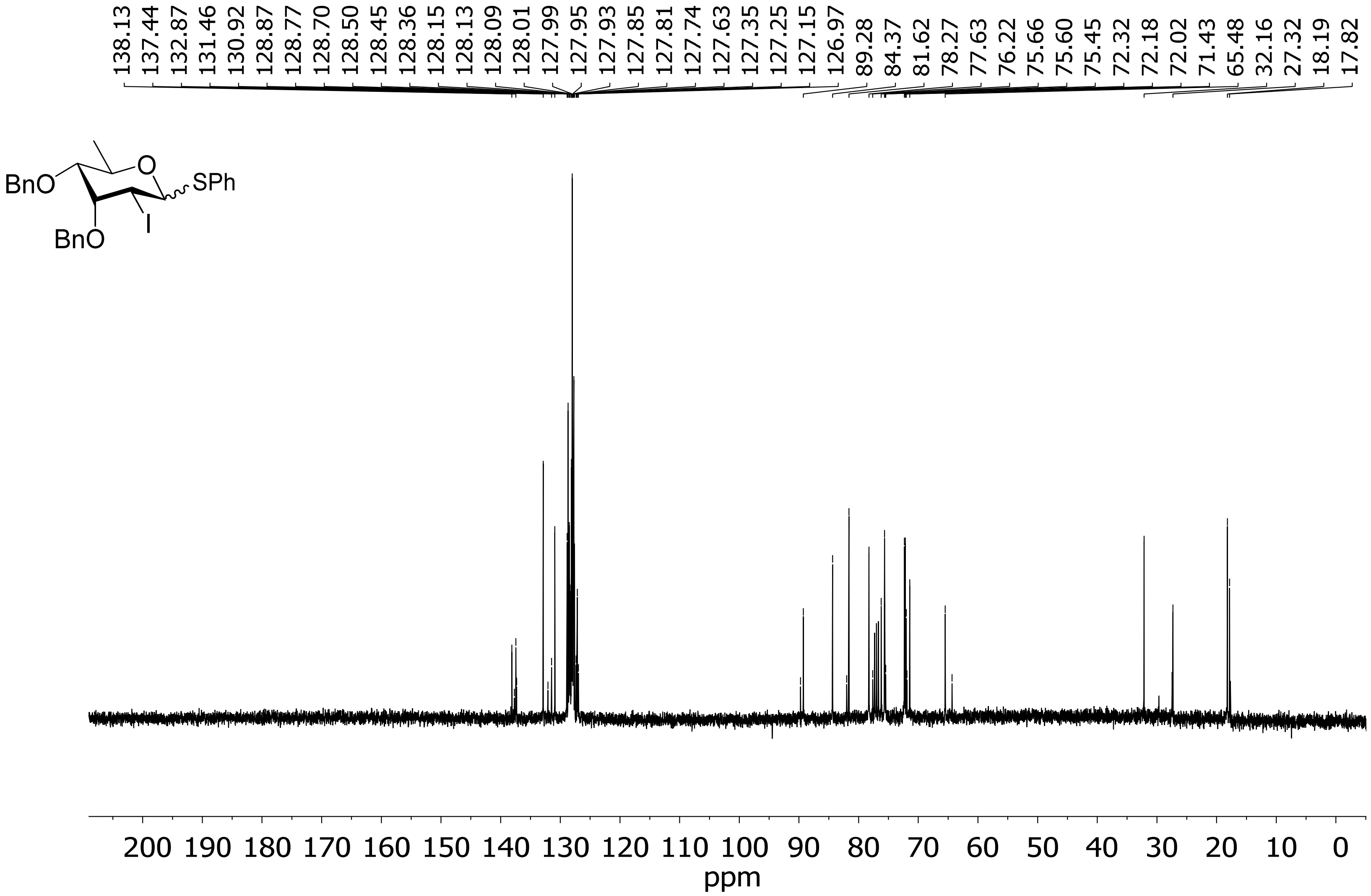


**Figure S8.** $^{13}C$ NMR ($CDCl_3$, 100.6 MHz) of **5b**

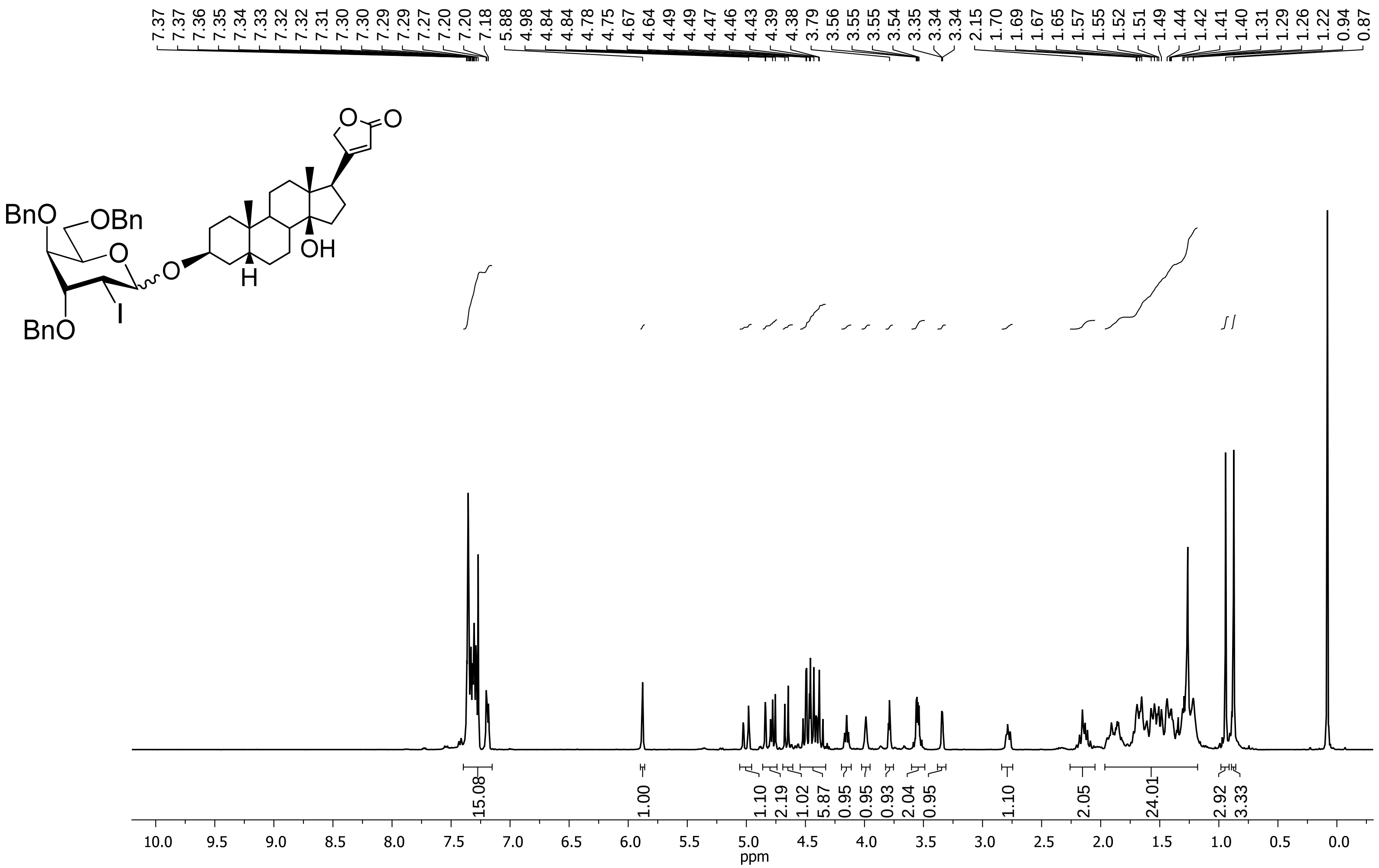

**Figure S9.** $^{1}$H NMR ($CDCl_3$, 400 MHz) of **7**

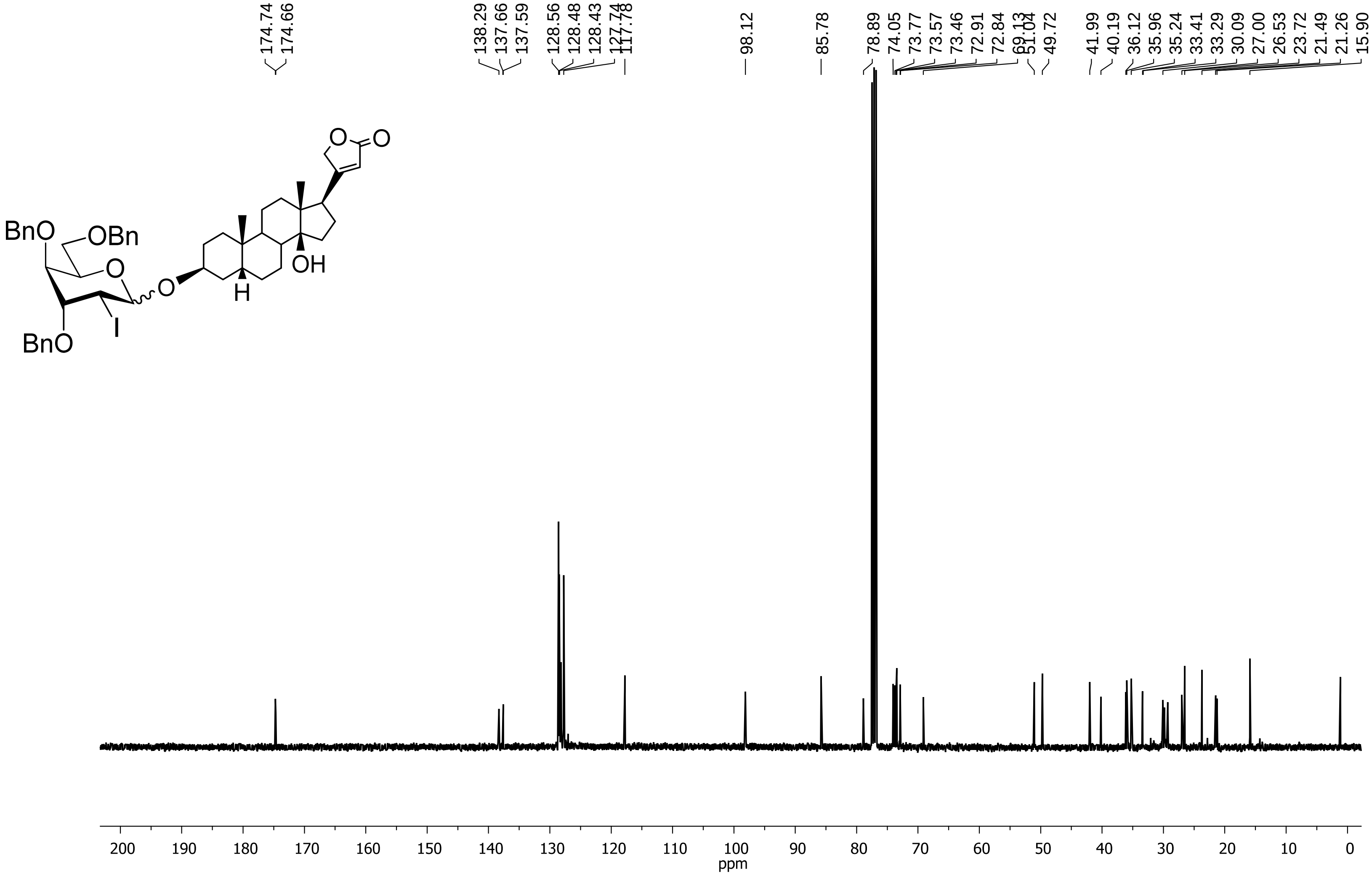


**Figure S10.** $^{13}C$ NMR ($CDCl_3$, 100.6 MHz) of **7**

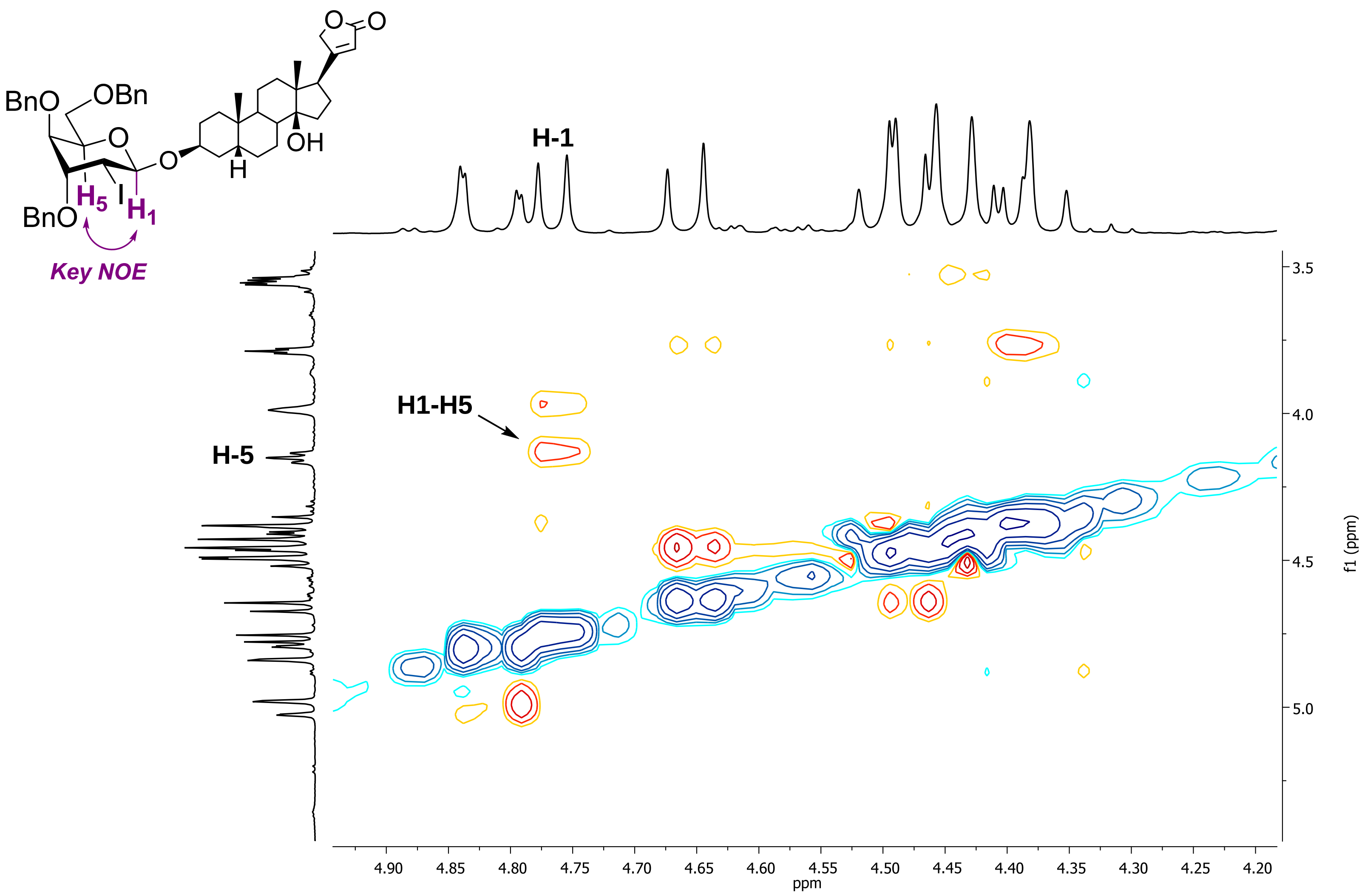


**Figure S11.** 2D NOESY ($CDCl_3$, 400 MHz) of **7**

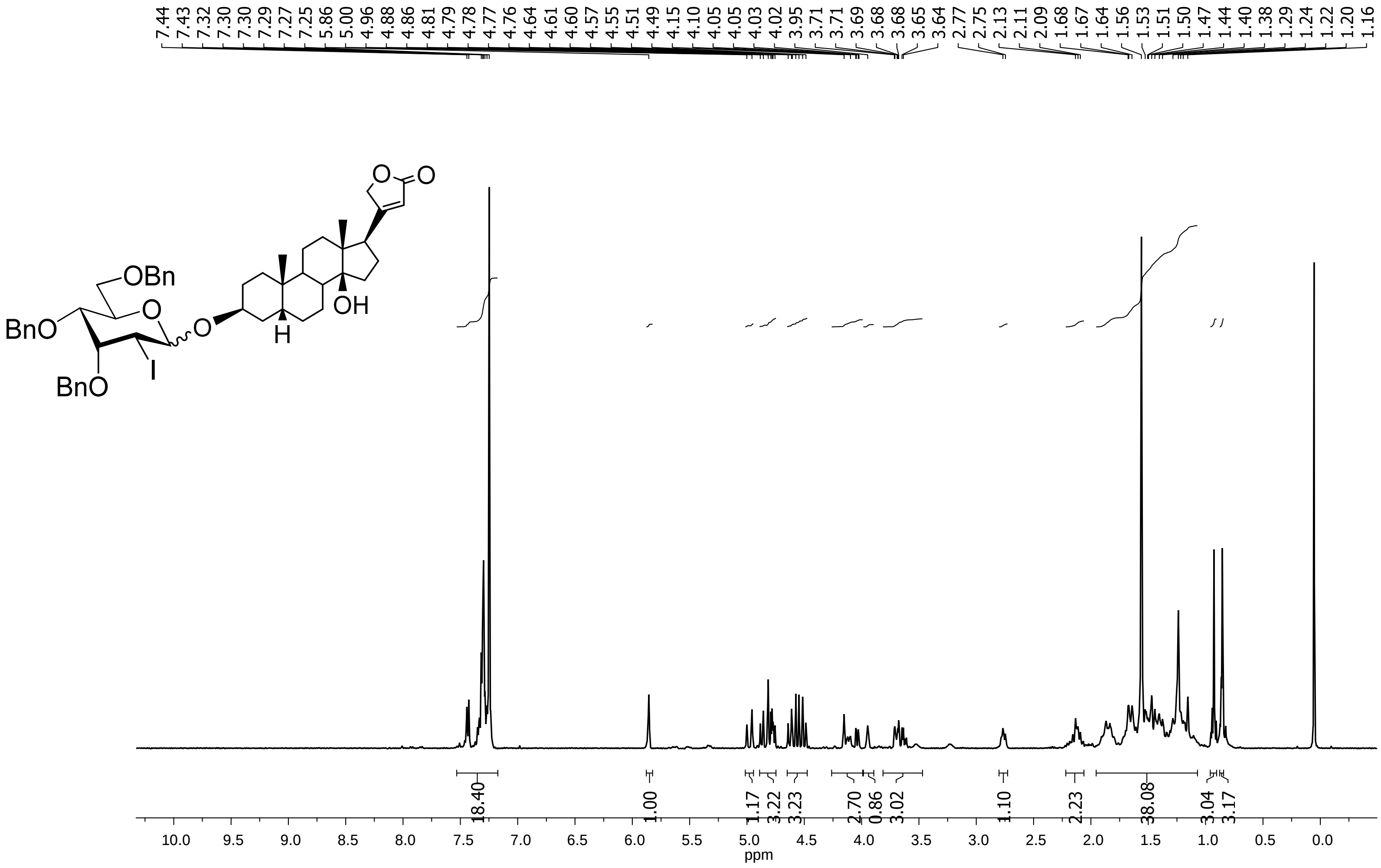

**Figure S12.** $^{1}$H NMR ($CDCl_3$, 400 MHz) of **8**

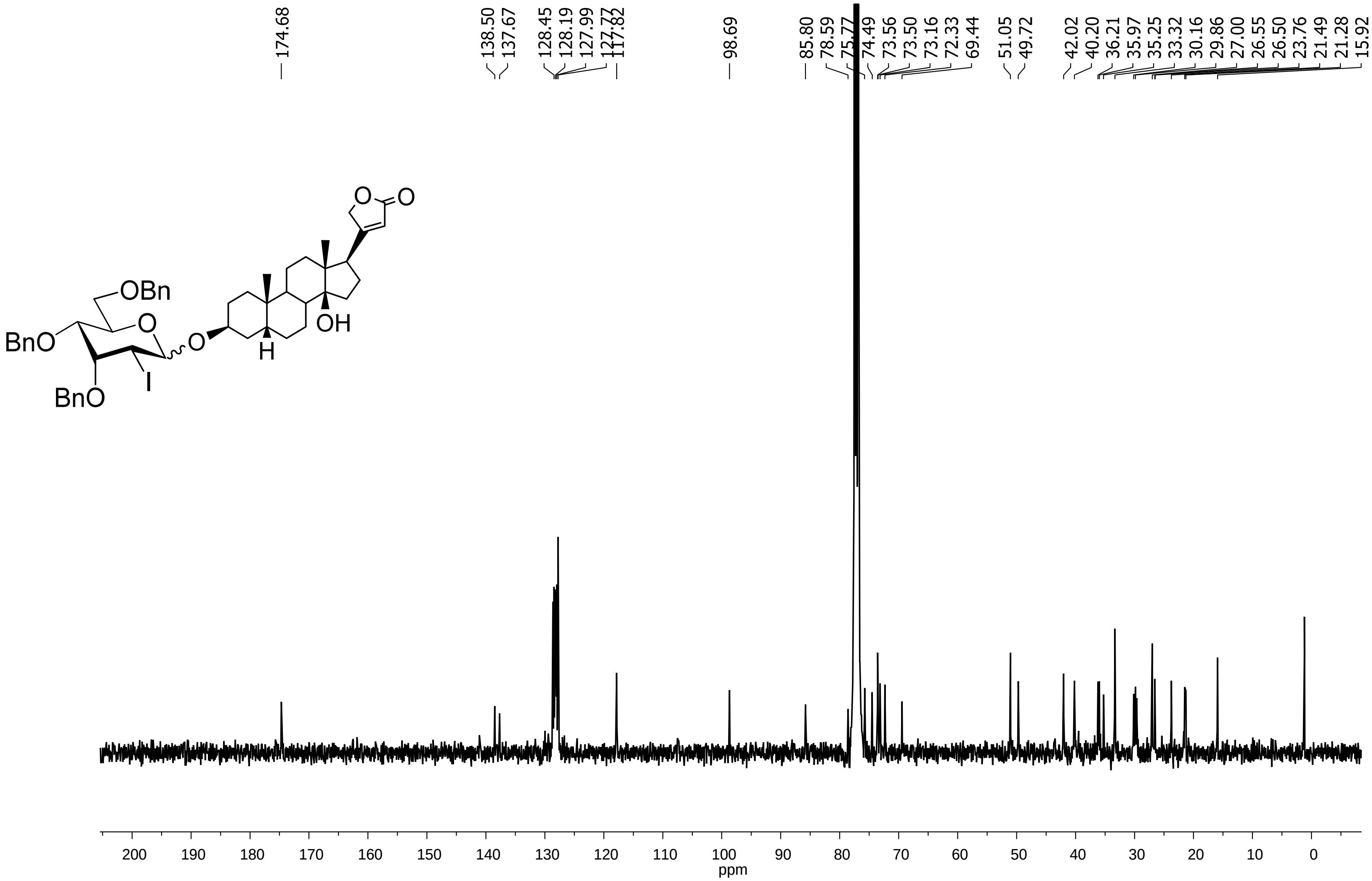


**Figure S13.** $^{13}$C NMR ($CDCl_3$, 100.6 MHz) of **8**

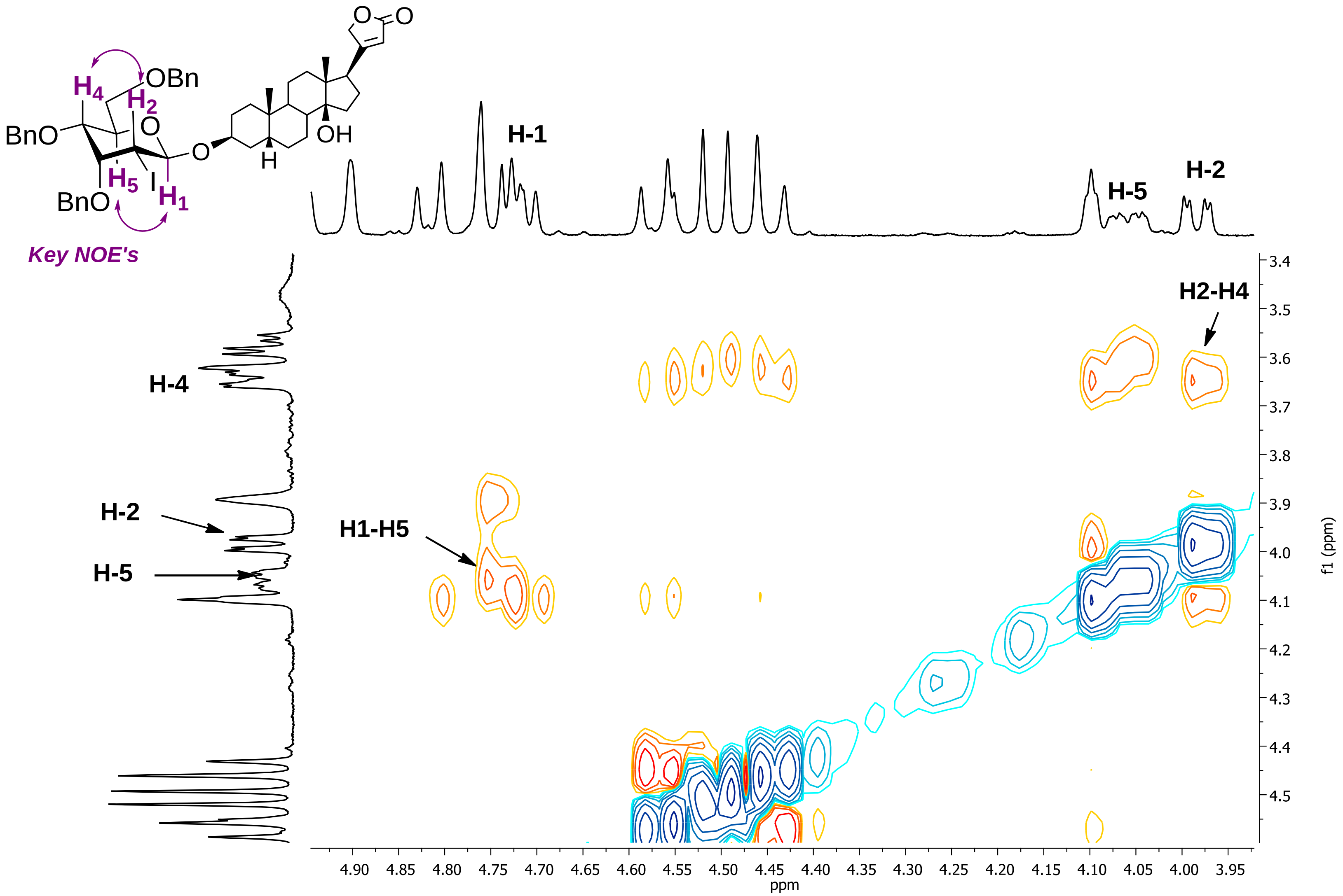


**Figure S14.** 2D NOESY ($CDCl_3$, 400 MHz) of **8**

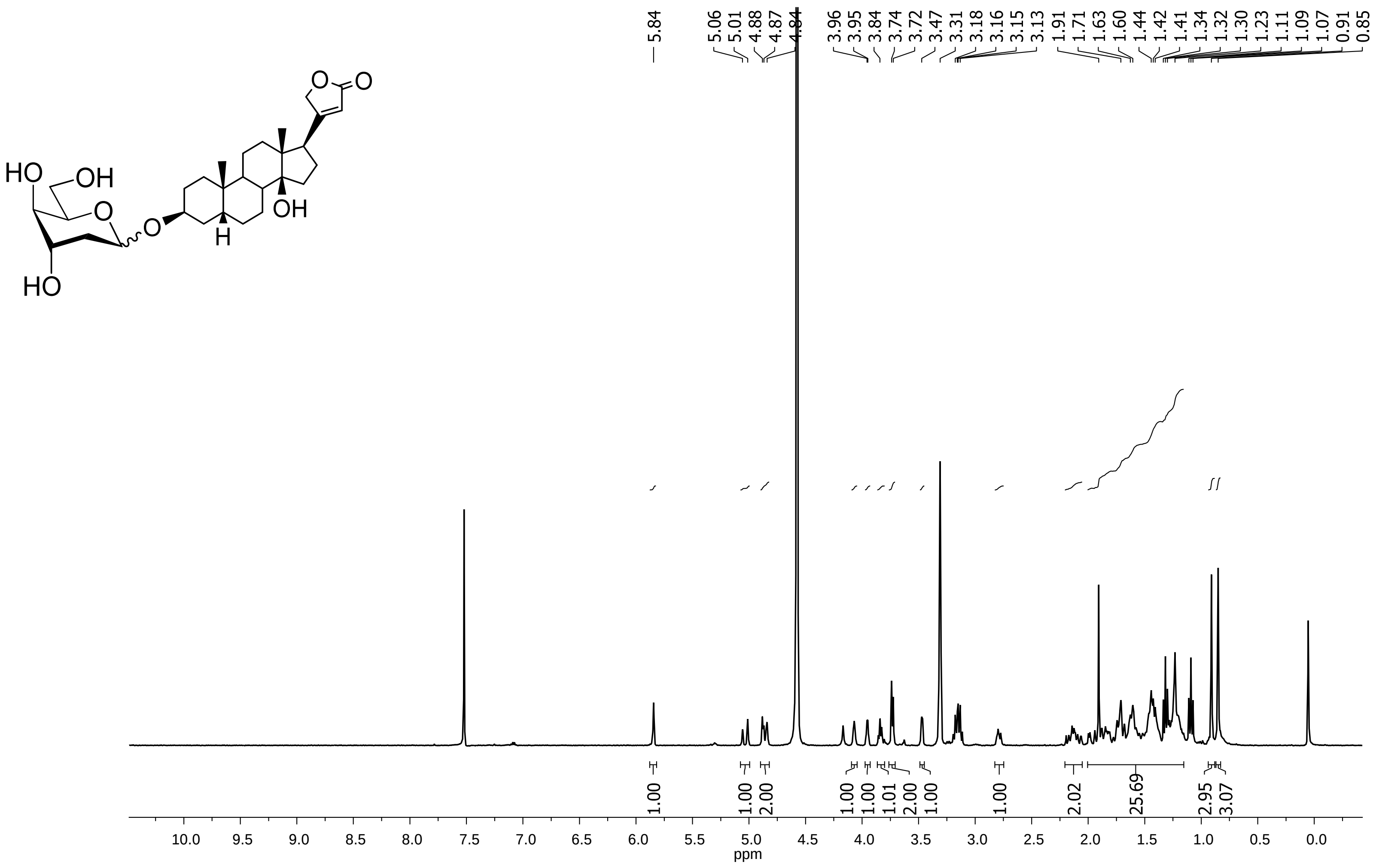

**Figure S15.** $^{1}H$ NMR (1:1 $CD_3OD/CDCl_3$, 400 MHz) of **9**

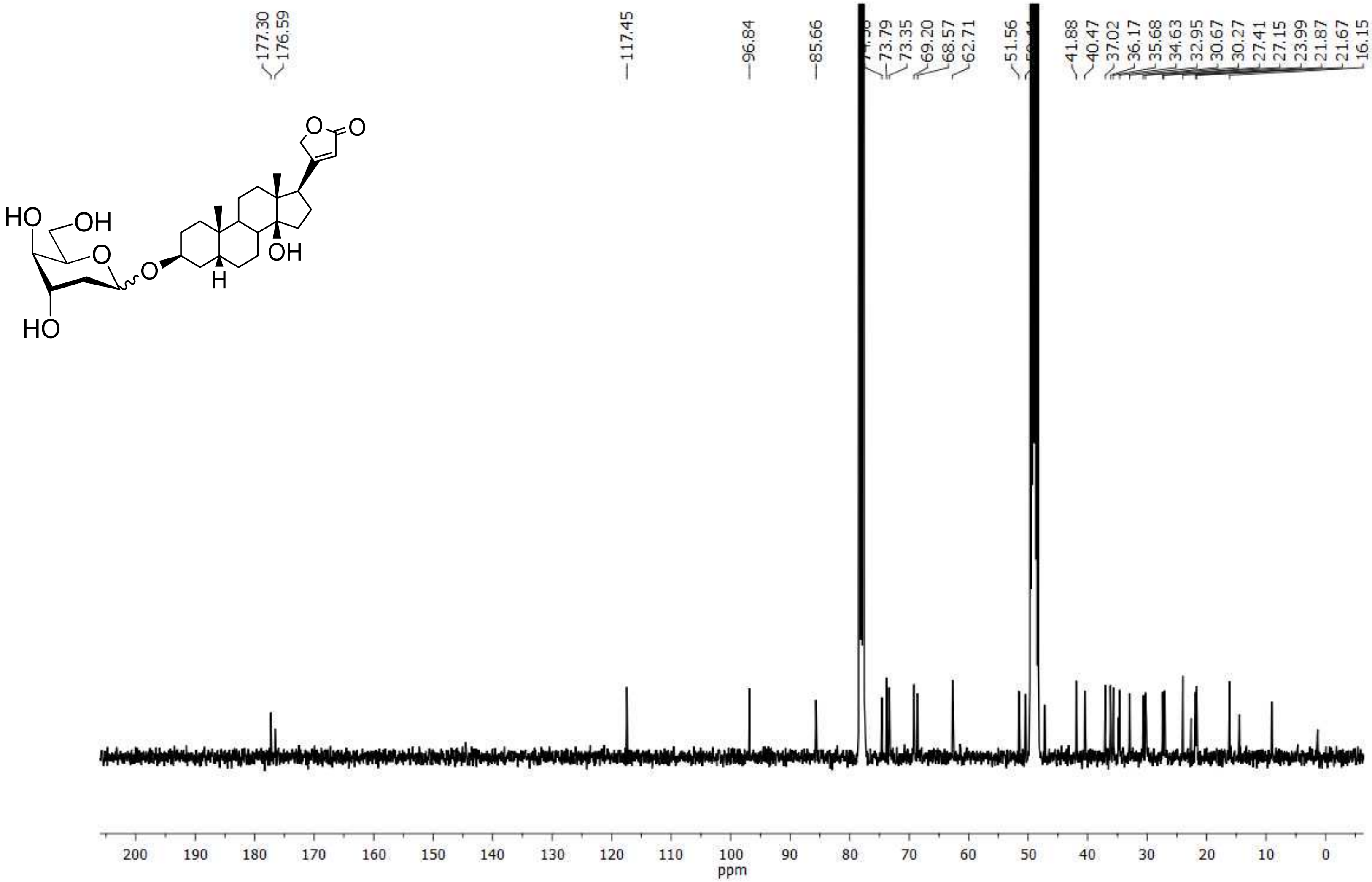


**Figure S16.** $^{13}C$ NMR (1:1 $CD_3OD/CDCl_3$, 100.6 MHz) of **9**

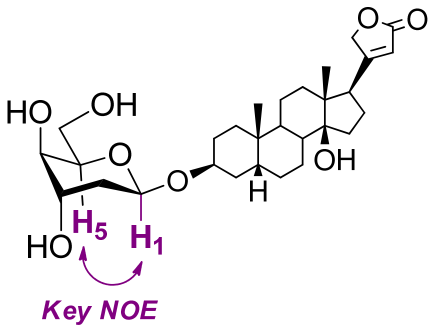


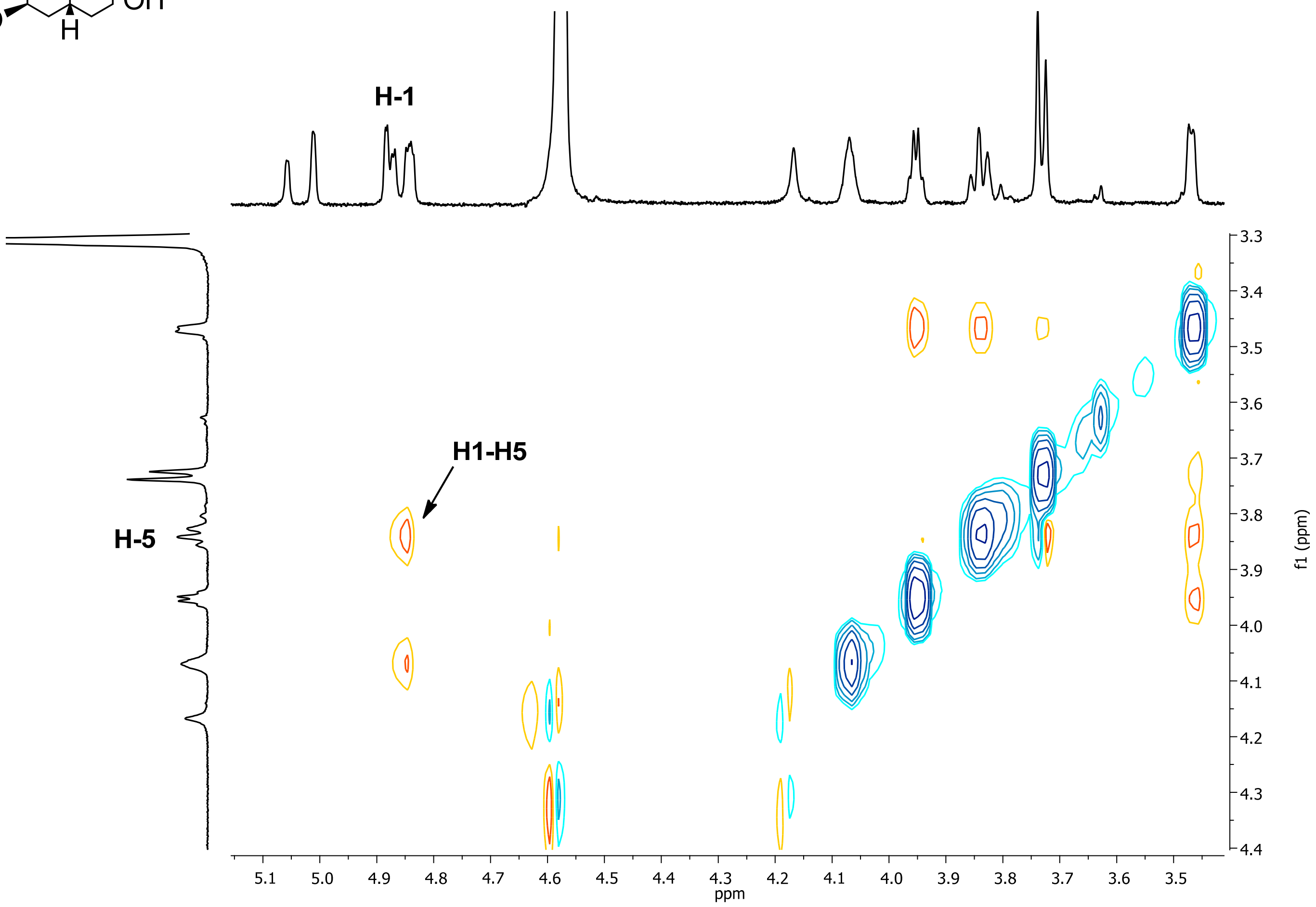


**Figure S17.** 2D NOESY (1:1 $CD_3OD/CDCl_3$, 400 MHz) of **9**

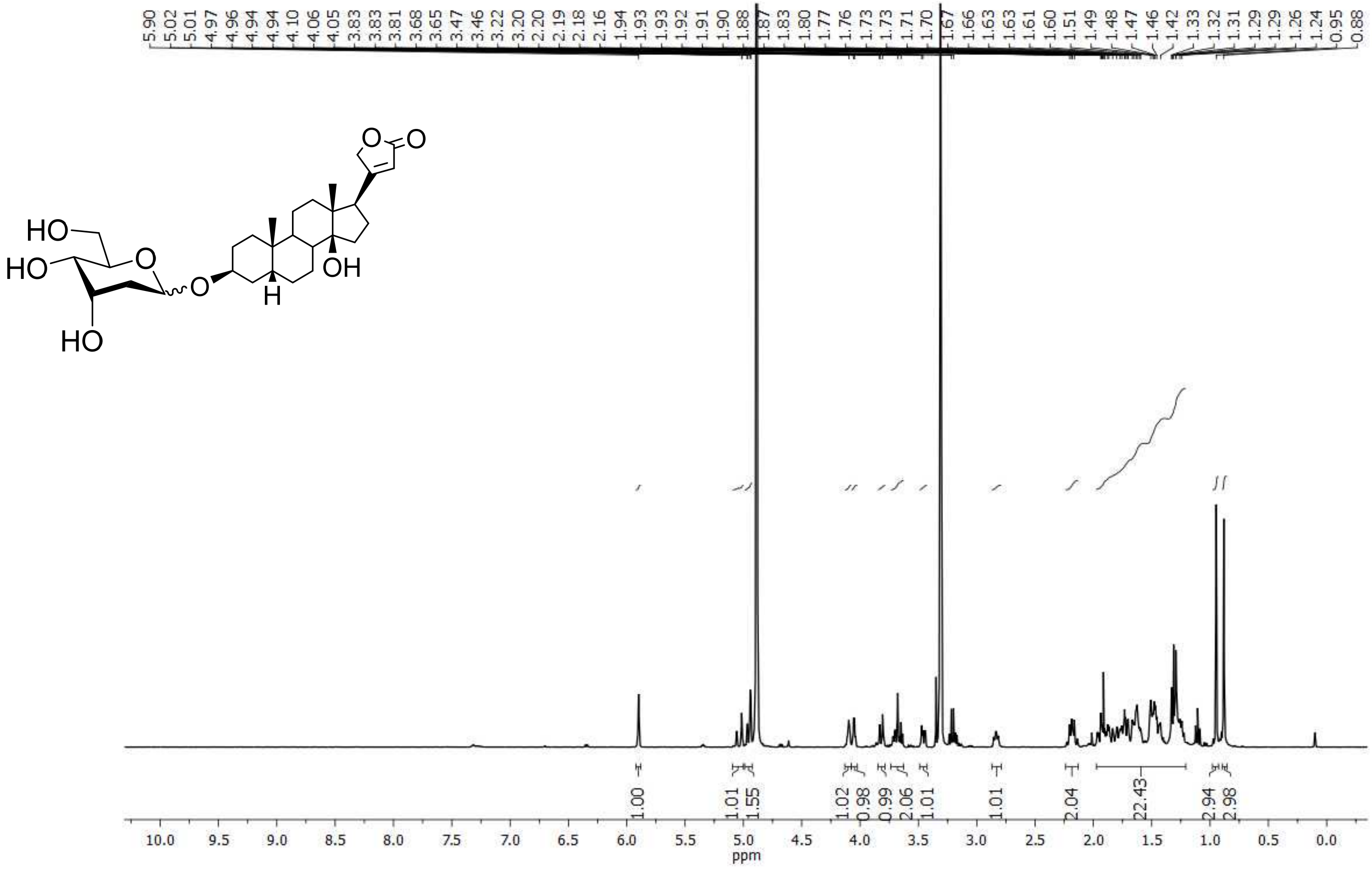

**Figure S18.** $^{1}H$ NMR ($CD_3OD$, 100.6 MHz) of **10**

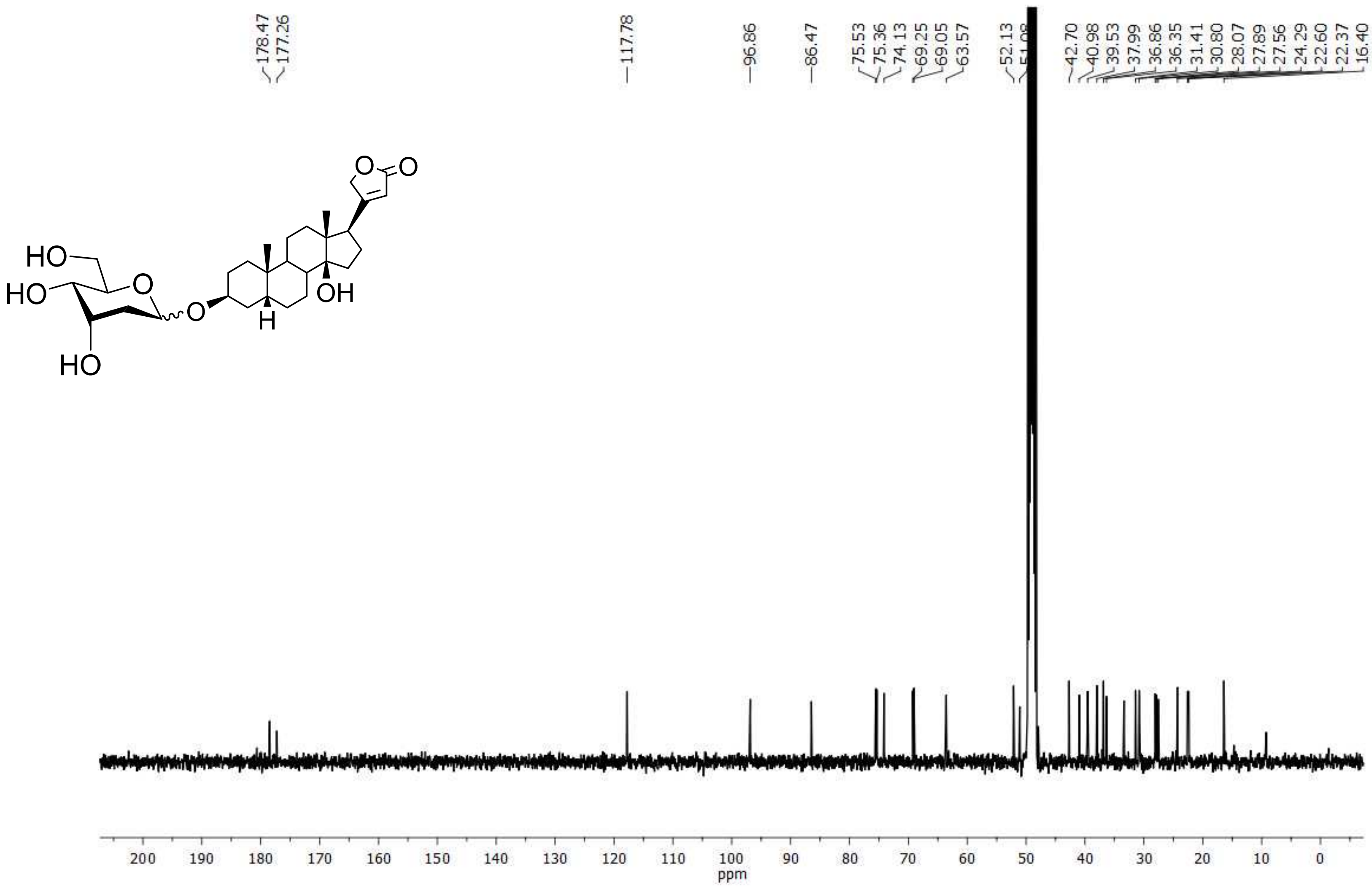


**Figure S19.** $^{13}$C NMR ($CD_3OD$, 100.6 MHz) of **10**

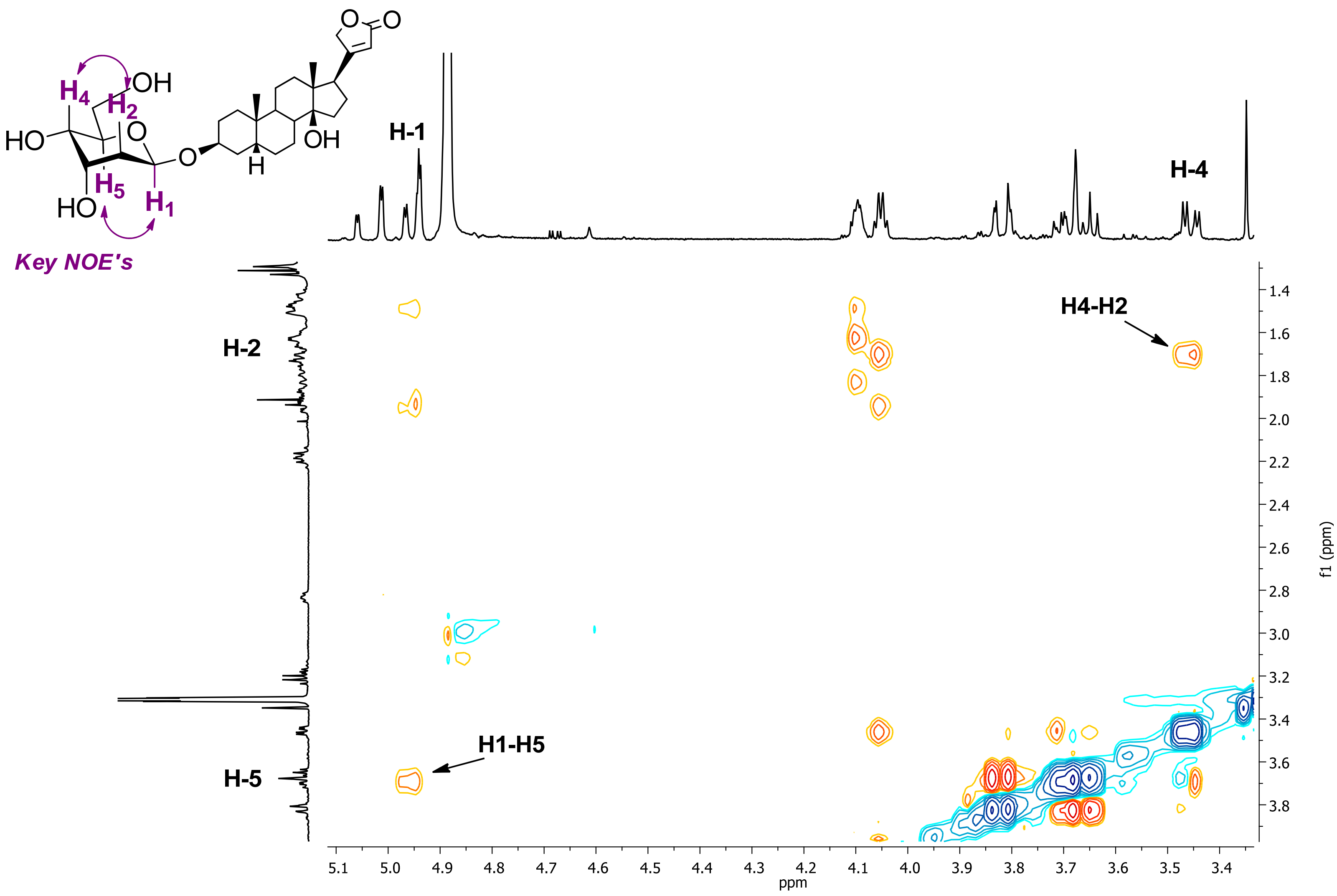


**Figure S20.** 2D NOESY ($CD_3OD$, 400 MHz) of **10**

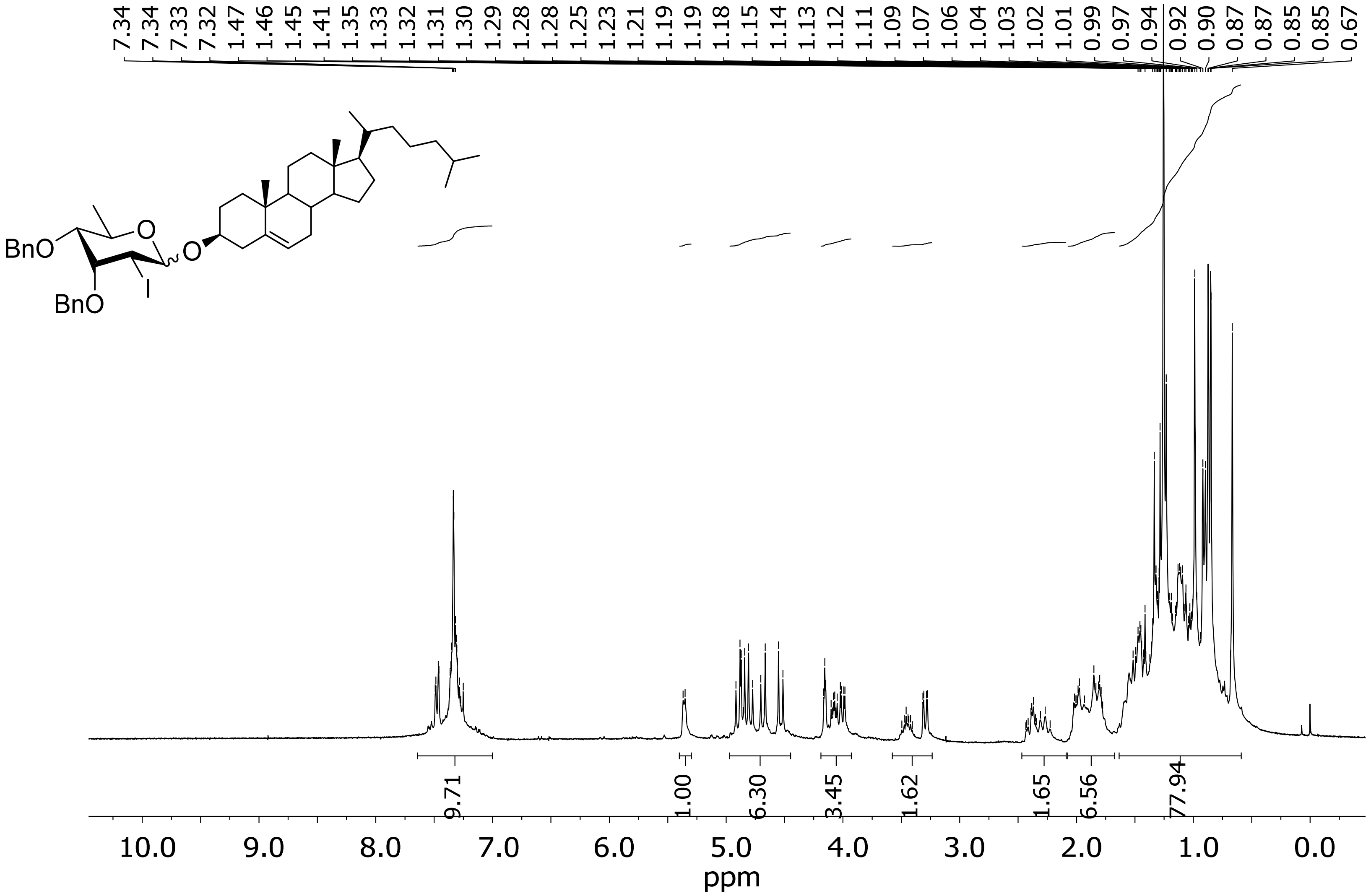


**Figure S21.** $^{1}H$ NMR ($CDCl_3$, 400 MHz) of **11**

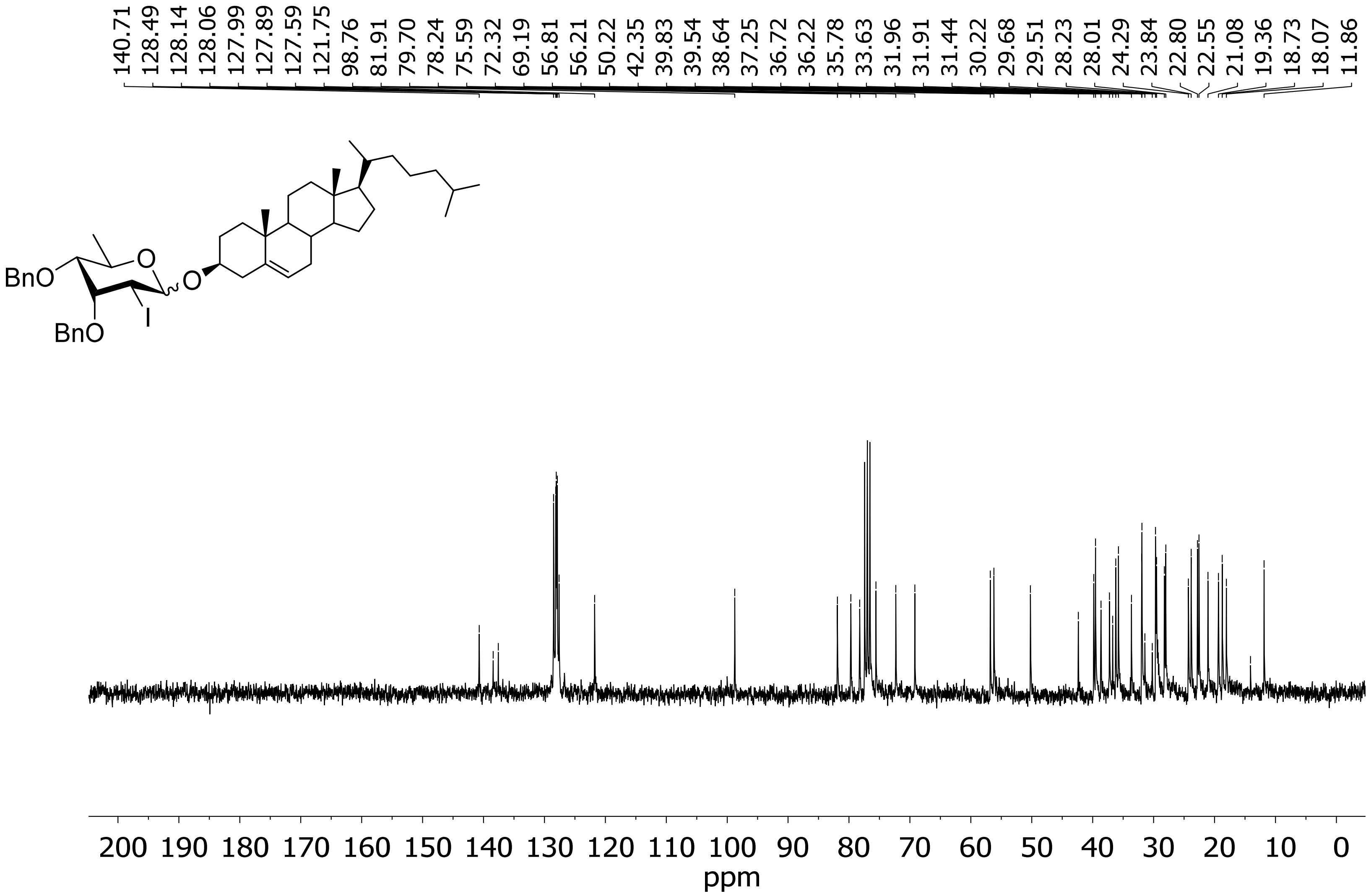


**Figure S22.** $^{13}$C NMR (CDCl$_3$, 100.6 MHz) of **11**
